# Soluble Frizzled-related proteins promote exosome-mediated Wnt re-secretion

**DOI:** 10.1038/s42003-024-05881-8

**Published:** 2024-03-01

**Authors:** Thi Hong Nguyen Tran, Ritsuko Takada, Elena Krayukhina, Takahiro Maruno, Yusuke Mii, Susumu Uchiyama, Shinji Takada

**Affiliations:** 1 Exploratory Research Center on Life and Living Systems (ExCELLS), National Institutes of Natural Sciences, 5-1 Higashiyama, Myodaiji-cho, Okazaki, Aichi 444-8787 Japan; 2 National Institute for Basic Biology, National Institutes of Natural Sciences, 5-1 Higashiyama, Myodaiji-cho, Okazaki, Aichi 444-8787 Japan; 3 The Graduate University for Advanced Studies (SOKENDAI), Okazaki, Aichi 444-8787 Japan; 4 U-Medico Inc., 2-1 Yamadaoka, Suita, Osaka 565-0871 Japan; 5 Graduate School of Engineering, Osaka University, 2-1 Yamadaoka, Suita, Osaka 565-0871 Japan; 6 PREST, Japan Science and Technology Agency (JST), Kawaguchi, Saitama 332-0012 Japan; 7 Present Address: Analytical Development Department, Chugai Pharmaceutical Co., Ltd., 5-5-1 Ukima, Kita-ku, Tokyo, 115-8543 Japan

**Keywords:** Extracellular signalling molecules, Protein translocation, Morphogen signalling, Secretion

## Abstract

Wnt proteins are thought to be transported in several ways in the extracellular space. For instance, they are known to be carried by exosomes and by Wnt-carrier proteins, such as sFRP proteins. However, little is known about whether and/or how these two transport systems are related. Here, we show that adding sFRP1 or sFRP2, but not sFRP3 or sFRP4, to culture medium containing Wnt3a or Wnt5a increases re-secretion of exosome-loaded Wnt proteins from cells. This effect of sFRP2 is counteracted by heparinase, which removes sugar chains on heparan sulfate proteoglycans (HSPGs), but is independent of LRP5/6, Wnt co-receptors essential for Wnt signaling. Wnt3a and Wnt5a specifically dimerize with sFRP2 in culture supernatant. Furthermore, a Wnt3a mutant defective in heterodimerization with sFRP2 impairs the ability to increase exosome-mediated Wnt3a re-secretion. Based on these results, we propose that Wnt heterodimerization with its carrier protein, sFRP2, enhances Wnt accumulation at sugar chains on HSPGs on the cell surface, leading to increased endocytosis and exosome-mediated Wnt re-secretion. Our results suggest that the range of action of Wnt ligands is controlled by coordination of different transport systems.

## Abstract (web summary)

Various carriers are involved in extracellular Wnt transport. Here, the authors show that Wnt heterodimerization with its carrier protein, sFRP2, enhances Wnt accumulation on the cell surface, leading to exosome-mediated Wnt re-secretion.

## Introduction

Secreted signaling proteins, or morphogens, such as Transforming growth factor β, Fibroblast growth factor (FGF), Hedgehog (Hh), and Wnt, participate in various aspects of embryogenesis. To understand regulatory mechanisms by morphogen signaling in embryogenesis, it is crucial to understand spatiotemporal details of morphogen transport. Although some morphogens are assumed to diffuse as monomers, various carriers have also been proposed for morphogen transport between producing and receiving cells^1,2^. For instance, secreted binding proteins may act as morphogen carriers by forming soluble protein complexes, as have been shown in the case of Scube2 for Hedgehog^3,4^. In addition, morphogen binding proteins on cell surfaces, like heparan sulfate proteoglycans (HSPGs), regulate release and trafficking of morphogens^5,6^. Extracellular membranous deliverers, like exosomes, also associate with many morphogens^7–16^. Furthermore, recent studies with imaging analyses revealed that intercellular transport of morphogens can be mediated by filopodium-like protrusions^17–28^. However, little is known about whether and/or how these different transport systems relate to one another.

Wnt is a secreted signal protein, post-translationally modified with palmitoleic acid ^29–31^. Therefore, in cultured cells, Wnt does not exist as a monomer unless detergent is added. Rather, Wnt requires carriers to shield hydrophobic lipid modification after secretion to the extracellular space. For instance, specific-Wnt binding proteins, including Swim in *Drosophila* and secreted Frizzled-related proteins (sFRPs) in vertebrates are reportedly involved in extracellular Wnt transport^32–34^. Furthermore, comprehensive size distribution analysis of GFP-Wnt3a revealed that secreted Wnt forms protein complexes, including high-molecular weight (HMW) complexes^33^, and heterodimers with a serum protein, afamin^35^. Notably, sFRP2, a member of the secreted Frizzled-Related Protein (sFRP) family, can dissociate HMW complexes and Wnt3a/afamin heterodimers, resulting in formation of Wnt3a/sFRP2 heterodimers, which can carry Wnt3a for long distances^33^. On the other hand, it has been suggested that various extracellular deliverers associated with Wnt are involved in shielding the hydrophobic modification with palmitoleic acid and Wnt trafficking. Extracellular membranous deliverers, including lipoprotein particles^36,37^ and exosomes^7–11,16,38^ also associate extracellularly with Wnt proteins. Since HSPGs are also involved in extracellular Wnt trafficking, it is likely that Wnt proteins associating with these carriers interact in some fashion with HSPGs on the cell surface during transport.

In addition to diversity of Wnt carriers, intracellular transport of Wnt appears to be complex. Several studies have shown that Wnt proteins secreted by the producing cell are endocytosed and then re-secreted. In the polarized epithelium of *Drosophila* imaginal discs, endocytosis is required for trafficking, secretion, and signaling of Wingless (Wg), the *Drosophila* ortholog of Wnt1^39^. Time-course analysis revealed that Wg is trafficked to the apical side, then internalized and re-released from the basolateral side for long-range diffusion in imaginal discs. On the other hand, it is suggested that Wnt is secreted via exosomes in a secondary release because this secretion is dependent on Ykt6^40,41^, a SNARE protein that recycles Wg to the plasma membrane.

In this study, using Wnt as a model, we addressed the question of whether various forms of morphogens are independently involved in their signaling in tissues or whether these forms affect one another in secretion and/or extracellular trafficking. In particular, we examined whether exosome-mediated re-secretion is affected by formation of Wnt complexes. To this end, we first established an assay system to detect re-secretion of Wnt3a on exosomes. Unexpectedly, we found that sFRP1 and sFRP2, but not sFRP3 or sFRP4, specifically enhance re-secretion of Wnt3a on exosomes. In addition, sFRP2 specifically increases cell surface attachment of Wnt3a in a manner dependent on HSPGs. Further analysis revealed that Wnt3a forms heterodimers with sFRP2, but not other sFRP members, in culture supernatant. Our results support the model that this specific interaction with sFRP2 appears to increase exosome-mediated re-secretion of Wnt3a by enhancing its attachment to HSPGs on the cell surface.

## Results

### sFRP2 increases exosomal re-secretion of Wnt3a

As a first step to examine the relationship between protein complex formation and secretion of Wnt proteins on exosomes, we examined the impact of several Wnt binding proteins on exosome-mediated secretion in our culture system (Supplementary Fig. 1a). To this end, L cells expressing GFP-tagged Wnt3a (GFP-Wnt3a) were co-cultured with HEK293 cells expressing the extracellular domain of Frizzled8 (exFzd8) or several sFRPs in a 9:1 ratio. The amount of Wnt3a secreted on exosomes was examined by ultracentrifugation, according to a previous study^16^. We examined exosomal Wnt3a by monitoring the amount of Wnt3a recovered in the pellet after ultracentrifugation of the co-culture supernatant at 100,000 x *g* (referred to as P100; Supplementary Fig. 1a). In this experimental condition, more that 95% of soluble GFP-Wnt3a secreted from L cells was recovered in the supernatant after ultracentrifugation at 100,000 x *g* (referred to as S100; Supplementary Fig. 1a-c).

As predicted, co-culture with exFzd8-expressing cells reduces the level of GFP-Wnt3a in the P100 fraction, suggesting that sequestration by exFzd8 reduces GFP-Wnt3a incorporation to exosomes (Supplementary Fig. 1b). To our surprise, co-culture with sFRP2-expressing cells resulted in a 20-fold increase in the amount of Wnt3a (Supplementary Fig. 1b, c). In contrast, co-culture with sFRP3 and sFRP4-expressing cells showed little effect on exosome-mediated secretion of Wnt3a (Supplementary Fig. 1b, d). Similar results were obtained using non-tagged Wnt3a (Supplementary Fig. 3a). Thus, sFRP2 specifically has a strong positive effect on Wnt3a secretion via exosomes.

**Fig. 1:**
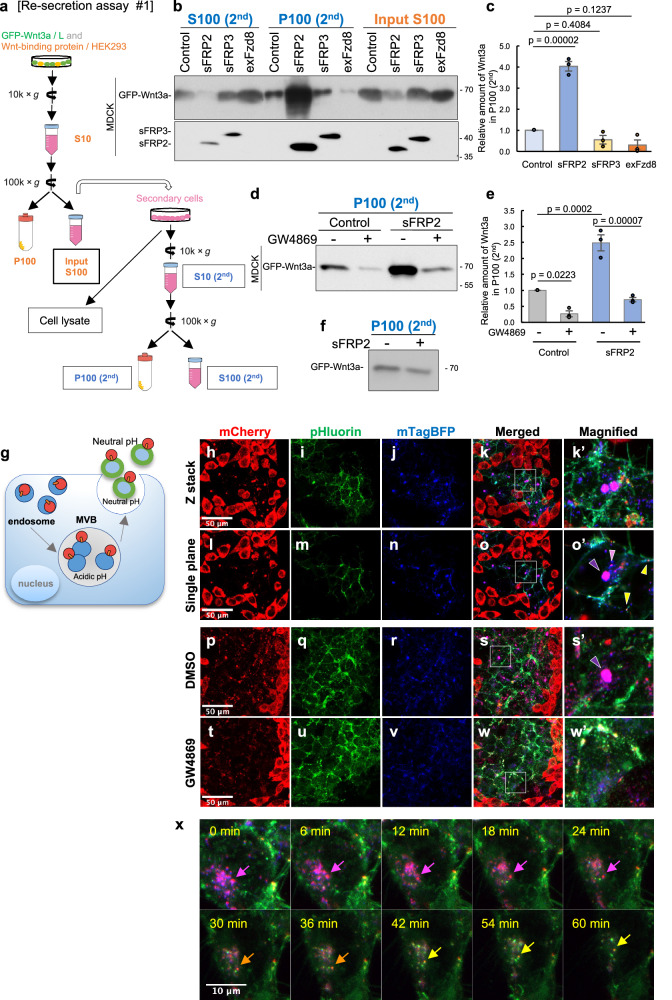
sFRP2 promotes Wnt3a secretion on exosomes. **a–c** Exosome-mediated re-secretion of GFP-Wnt3a from MDCK cells. The experimental procedure, referred to as Re-secretion assay #1, is shown in (**a**). At 24 h after treatment with exosome-depleted S100 supernatant (input S100) prepared from co-culture of GFP-Wnt3a/L and HEK293 cells with or without producing sFRP2, sFRP3, or exFzd8, amounts of re-secreted GFP-Wnt3a from MDCK cells were examined by Western blotting after fractionation into S100 (S100 (2nd)) and P100 (P100 (2nd)) fractions by ultracentrifugation (**b**). In (**b**), each membrane was blotted separately. Relative amounts (amount in P100 (2nd) / amount in input S100) of GFP-Wnt3a shown in (**b**) were examined as shown in (**c**). **d**, **e** The effect of MVB inhibitor on exosome-mediated re-secretion of GFP-Wnt3a. MDCK cells cultured with exosome-depleted CS (input S100) prepared from co-culture of GFP-Wnt3a cells and sFRP2-expressing cells were treated without inhibitor or with MVB inhibitor GW4689. Amounts of GFP-Wnt3a were examined as shown in (**e**). In (**b**, **d**), S100 (2nd) and/or P100 (2nd), were subjected to Western blot to examine relative amounts of GFP-Wnt3a loaded on exosomes. Relative amounts of GFP-Wnt3a shown in (**c**, **e**) were quantified using Image J software and results are shown as mean ± s.e. Statistical significance was calculated by one-way ANOVA or two-way ANOVA followed by Turkey HSD test, respectively, n = 3 independent experiments. p < 0.05 was considered statistically significant. **f** Examination of whether sFRP2 increases the amount of GFP-Wnt3a under cell-free conditions. The experimental procedure is shown in Supplementary Fig. 7. Briefly, exosome-depleted S100 of GFP-Wnt3a/L and that of sFRP2-expressing HEK293 or control HEK293 was mixed with S10 of parental MDCK cells, from which exosomes had not yet been removed and still contained exosomes. These were further incubated for 24 h at 37 ^o^C, followed by P100 fractionation. **g–o** Distribution of mCherry-Wnt3a and pHluorin-M153R-CD63-mTagBFP in sFRP2-expressing cells. To examine localization and movement of Wnt3a after incorporation into secondary cells, mCherry-Wnt3a producing L cells were co-cultured with HEK293 cells in which sFRP2 and pHluorin-M153R-CD63-mTagBFP were expressed. Since pHluorin is not fluorescent in acidic conditions, like cytoplasm, intracellular (BFP only) and extracellular (double-positive BFP and GFP) CD63 can be detected by different colors (**g**). Cells were inoculated 1 day before observation. mCherry-Wnt3a images (**h**, **l**), extracellular CD63 images detected by pHluorin (**i**, **m**), total CD63 images detected by mTagBFP (**j**, **n**), and merged images (**k**, **o**) of mCherry-Wnt3a and intracellular and total CD63 are shown. Z-stacked images (**h**–**k**) and images at single confocal planes (**l–o**) are shown. Magnified images of the area surrounded by white lines in (**k**, **o**) are indicated in (**k’**, **o’**), respectively. Of note, most incorporated mCherry-Wnt3a coexisted with intracellular CD63 in large puncta (purple arrowhead), probably MVB and small puncta (pink arrowhead), probably endosomes. In addition, extracellular coexistence of mCherry-Wnt3a and CD63 was detected (mCherry, pHluorin and mTagBFP triple-positive puncta; indicated by yellow arrowheads). **p–w** Effect of MVB inhibitor to distribution of mCherry-Wnt3a and pHluorin-M153R-CD63-mTagBFP. Distribution of mCherry-Wnt3a and pHluorin-M153R-CD63-mTagBFP was examined with or without MVB inhibitor, GW4869. The experimental procedure was the same as that shown in (**h**)–(**k**) except for addition of GW4869. Scale bar; 50 μm. **x** Time-lapse analysis of mCherry-Wnt3a puncta in pHluorin-M153R-CD63 expressing cells with 6-min intervals from Supplementary Movie 1. Note that mCherry-Wnt3a coexisting with CD63 gradually changes color, showing that mCherry-Wnt3a is re-secreted with CD63 (arrows). Scale bar; 10 μm.

As described in the Introduction, in the imaginal disc of *Drosophila*, it has been proposed that secreted Wnt proteins are taken up by cells and then released again into the extracellular space^40–42^. Furthermore, Wnt re-secretion is dependent on an endosomal recycling mechanism required for exosome-mediated secretion^40,41^. To examine whether sFRP2 affects this re-secretion process, we established an assay system to investigate exosomal re-secretion of Wnt (Fig. 1a). First, exosome-depleted culture supernatant (CS) was prepared by ultracentrifugation at 100,000 x *g* from L cells expressing GFP-Wnt3a. Then, secondary cells were treated with this supernatant to follow endocytosis of Wnt3a and release of Wnt3a via exosomes. We refer to this protocol, in which exosomal re-secretion of Wnt can be examined, as Re-secretion assay #1. In this protocol, as well as Re-secretion assay #2, shown later, the amount of Wnt3a re-secreted on exosomes was monitored by recovery in the pellet after ultracentrifugation of the culture supernatant at 100,000 x *g* (referred to as P100; Fig. 1a). Release of exosome-mediated Wnt3a by monitoring GFP-Wnt3a in the P100 fraction was detectable 24 h after addition of GFP-Wnt3a CS (Fig. 1b, c). This release was actually decreased by treatment with GW4869, a chemical that inhibits formation of multivesicular bodies (MVB; Fig. 1d, e)^43,44,45^. Since endocytosed proteins are incorporated into exosomes via MVBs, this result indicates that this method monitors exosome-mediated re-secretion of Wnt ligands.

Then, we examined the effect of soluble Wnt-binding proteins on exosome-mediated re-secretion of Wnt3a by examining recovery in the P100 fraction (Fig. 1b)^38,46^. MDCK cells were treated with exosome-depleted CS (input S100 fraction) of the co-culture of GFP-Wnt3a-expressing L cells and HEK293 cells with expression of sFRP2, sFRP3, or exFzd8, as well as sFRP1. As predicted, CS prepared from co-culture with exFzd8-expressing cells reduced the level of GFP-Wnt3a in the P100 fraction, suggesting that sequestration by exFzd8 reduces GFP-Wnt3a incorporation into exosomes (Fig. 1b). To our surprise, CS from co-culture with sFRP1- and sFRP2-expressing cells resulted in an apparent increase in the amount of Wnt3a (Supplementary Fig. 2, Fig. 1b, c), whereas that with sFRP3-expressing cells showed little effect on exosome-mediated re-secretion of Wnt3a (Fig. 1b). Because this effect of sFRP2 was also sensitive to GW4869 (Fig. 1d, e), sFRP2 likely impacts exosome-mediated re-secretion of Wnt3a. Similar results were obtained using non-tagged Wnt3a (Supplementary Fig. 3b).

This effect of sFRP2 was also detected when we prepared GFP-Wnt3a and sFRP2 from separate cultures (Re-secretion assay #2) (Supplementary Fig. 4a-c). Several cell lines, including MDCK, L and HEK293, were used as secondary cells (Supplementary Fig. 5). Time course analysis with L cells showed that the amount of exosomal Wnt3a gradually increased after addition of exosome-depleted CS from control and sFRP2-expressing cells (Supplementary Fig. 6a). We observed an sFRP2-dependent increase of exosome-mediated Wnt3a re-secretion (Supplementary Fig. 5, 6a). In addition to the amount of re-secreted exosomal GFP-Wnt3a, its signaling activity was actually increased by sFRP2 at 24 h after addition of CS (Supplementary Fig. 6b). The effect of sFRP2 was detected with or without a GFP tag (Supplementary Fig. 4b, c), but CS from sFPR3, sFRP4 and exFzd8-expressing cells did not increase exosomal re-secretion of Wnt3a. Thus, interaction between Wnt3a and sFRP2 after secretion from producing cells causes increased Wnt3a re-secretion on exosomes. In contrast, simple incubation of exosome-depleted CS (input S100 fraction) of GFP-Wnt3a-expressing cells and that of sFRP2-expressing cells with exosome-containing medium, S10 of parental MDCK, did not increase the amount of Wnt3a in the P100 fraction (Fig. 1f, Supplementary Fig. 7), further suggesting that the effect of sFRP2 is cell-mediated, i.e., endocytosis and MVB-mediated re-secretion.

Next, we directly visualized movement of Wnt3a after incorporation into cells (Fig. 1g–x). Movement of mCherry-Wnt3a was examined by comparing with CD63 fused with BFP and pH-sensitive green fluorescent protein, pHluorin. Since pHluorin is not detectable at low pH, as in cytoplasm, but is detectable after secretion, we can distinguish intracellular and extracellular CD63 by fluorescence (Fig. 1g–x)^47^. Given that CD63, an exosome marker, is also enriched in the exosome secretion pathway, including MVB, this tool allows us to judge incorporation of Wnt3a into the exosome secretion pathway (Fig. 1g). In this experiment, HEK293 cells expressing BFP and pHluorin-fused CD63 were surrounded by mCherry-Wnt3a expressing L cells and sFRP2 was supplied by expression in fluorescent CD63-expressing HEK293 cells. Strikingly, under these conditions, most incorporated mCherry-Wnt3a was co-localized with cytosolic CD63 (Fig. 1k, o, k’, o’), which is detectable only by BFP fluorescence. In particular, many mCherry-Wnt3a proteins were assembled in huge CD63-positive cytosolic structures (Fig. 1o’). These structures appeared to be MVBs because they were sensitive to MVB inhibitor, GW4869 (Fig. 1p-w). In addition, the presence of many small cytosolic CD63 and mCherry-Wnt3a double-positive vesicles suggests that mCherry-Wnt3a is trafficked via CD63-positive endosomes (Fig. 1o’). Furthermore, exosomal re-secretion of mCherry-Wnt3a was also detectable because a small amount of mCherry-Wnt3a protein was co-localized with extracellular CD63, which was detectable by pHluorin fluorescence (Fig. 1o’). Time lapse imaging analysis showed the color change of CD63 and mCherry-Wnt3a double-positive puncta from magenta to yellow, indicating that the double-positive intracellular puncta were actually secreted (Fig. 1x, Supplementary Fig. 8 and 9, Supplementary Movie 1). Taken together, sFRP2 specifically increases re-secretion of Wnt3a on exosomes in various cell lines.

### sFRP2 increases cell surface attachment of Wnt3a

**Fig. 2:**
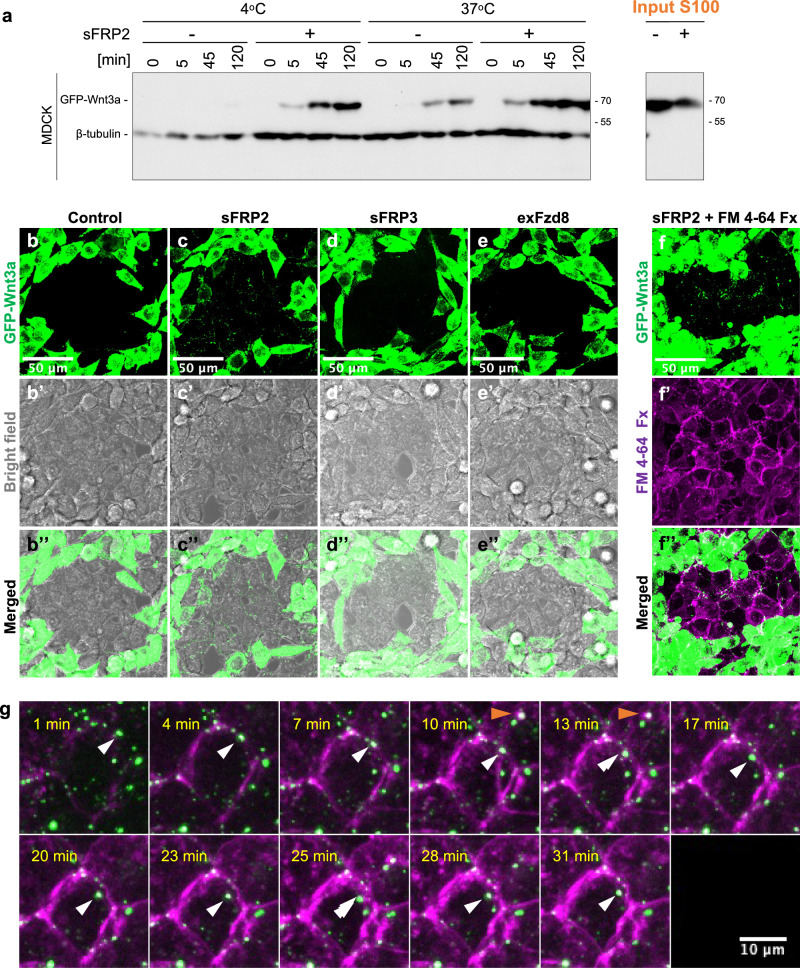
sFRP2 specifically promotes attachment of Wnt3a on cell surfaces. **a** Western blot detection of GFP-Wnt3a in lysates of MDCK cells collected at different times after treatment of the CS of co-culture of GFP-Wnt3a/L cells with sFRP2-expressing or control HEK293 cells. Experiments were carried out at 4 °C or 37 °C. β-tubulin was detected as an internal control. **b–f** Visualization of GFP-Wnt3a in sFRP2-, sFRP3-, exFzd8-expressing, or control cells. To examine Wnt3a distribution in the presence of sFRP2 sFRP3, or exFzd8, GFP-Wnt3a-producing L cells were co-cultured with sFRP2-, sFRP3-, or exFzd8-expressing or control HEK293 cells for 2 days before observation. All images were processed by maximum intensity projection. Three independent sets were observed for each co-culture combination. GFP fluorescence images (**b–e**), bright field images (**b’–e’)**, and their merged images (**b”–e”**) are shown. Images in the area where sFRP, exFzd8-expressing or control cells were surrounded by GFP-Wnt3a-expressing cells are shown. In addition, FRP2-expressing cells and surrounding GFP-Wnt3a/L cells were stained with FM 4–64 Fx, a lipophilic probe that fluoresces upon binding to cell membranes and quickly endocytosed (GFP-Wnt3a image in (**f**), FM 4–64 image shown in magenta in (**f’**), and merged image in (**f”**)). Note that many GFP-Wnt3a puncta are localized along the cell surface. Analysis of XZ planes also revealed that some green puncta in **f”** are incorporated into cells (Supplementary Fig. 11). Scale bar; 50 μm. **g** Time-course images of GFP-Wnt3a co-stained with FM 4–64 at indicated time-point from Supplementary Movie 3. The movement of GFP fluorescent puncta with FM 4–64 in sFRP2-expressing HEK293 cells was tracked. In (**f**, **f’**, **f”**), cells were fixed 1 min after treatment with FM 4–64. Many green puncta overlap with FM 4–64 signals following a short treatment. White and orange arrowheads in (**g**) indicate movement of GFP-Wnt3a puncta incorporated into cells. Scale bar; 10 μm.

To investigate how sFRP2 increases re-secretion of Wnt3a on exosomes, we next examined amounts of GFP-Wnt3a proteins in lysates of secondary cells after treatment with exosome-depleted CS (input S100 fraction) according to Re-secretion assay #1 (Fig. 1a) or #2 (Supplementary Fig. 4a). The amount of GFP-Wnt3a was clearly increased in MDCK (Fig. 2a; Supplementary Fig. 10b, c), L (Supplementary Fig. 10a, c), and HEK293 cells (Supplementary Fig. 10c) in the presence of sFRP2 at 37 °C. However, because sFRP2 increased the amount of GFP-Wnt3a even at 4 °C (Fig. 2a, Supplementary Fig. 10a), this increase was not primarily due to an increase in endocytosis. Rather, this result shows that the increase already occurred before endocytosis, probably at attachment of GFP-Wnt3a to cell surfaces.

We also examined the effect of sFRP2 on distribution of Wnt3a using light microscopy. When sFRP2-expressing HEK293 cell colonies were surrounded by GFP-Wnt3a-expressing L cells, many GFP-positive puncta were clearly localized along cell boundaries of sFRP2-expressing cells (Fig. 2c, f). In contrast, this boundary localization was scarcely observed when control HEK293 and sFRP3-, exFzd8-expressing cells were used instead of sFRP2-expressing cells (Fig. 2b, d, e). Furthermore, by time-lapse imaging, movement of GFP puncta from cell surfaces into cells was observed in sFRP2-expressing HEK293 cells, showing that endocytosis of GFP-Wnt3a occurs specifically in these cells (Fig. 2c, f, g; Supplementary Movie 2, 3, Supplementary Fig. 11). These biochemical and imaging results suggest that sFRP2 enhances association of Wnt3a with cell surfaces, which likely follows an increase in endocytosis.

### Membrane proteins are involved in Wnt3a re-secretion

**Fig. 3:**
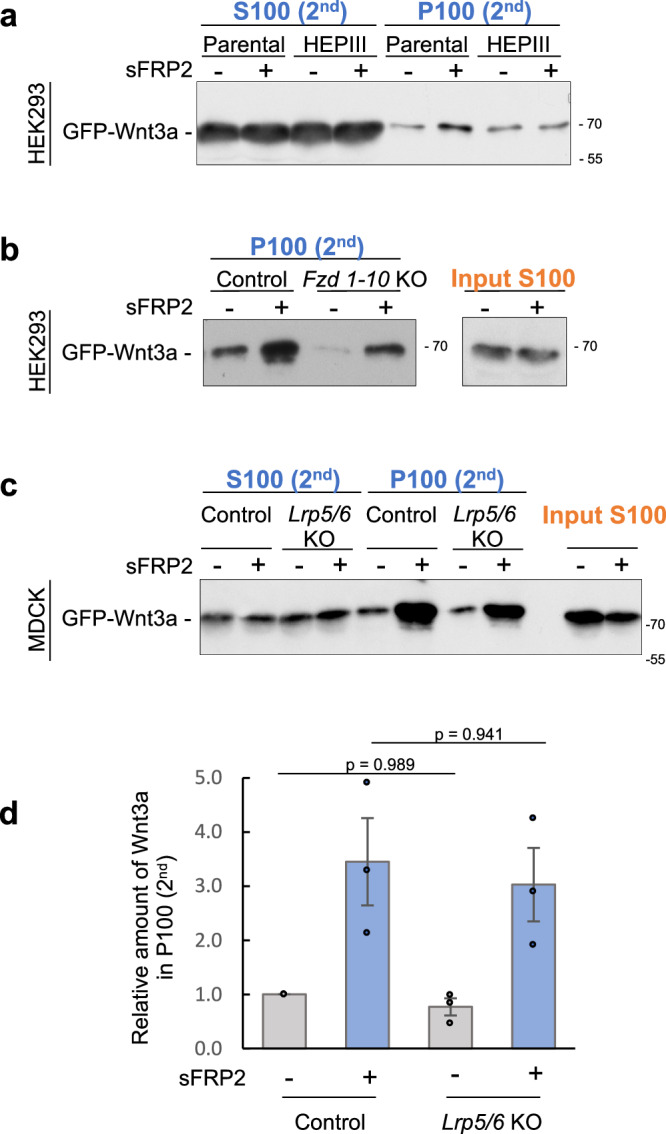
Effect of membrane proteins on sFRP2-induced re-secretion of GFP-Wnt3a in exosomes. **a** Effect of heparinase in sFRP2-induced exosome-mediated secretion of GFP-Wnt3a. Western blot analysis of GFP-Wnt3a recovered in exosome-enriched P100 (2nd) fraction, as well as S100 (2nd) collected 24 h after treatment of exosome-depleted CS from co-culture of GFP-Wnt3a-expressing cells and sFRP2-expressing or control cells in parental or Heparinase III (HepIII) expressing HEK293 cells. **b-d**. Examination of involvement of receptors of Frizzled family proteins (**b**) and co-receptors of LRP5 and LRP6 (**c**, **d**) in the increase of exosome-mediated GFP-Wnt3a re-secretion by sFRP2. *Fzd 1–10* KO and control HEK293 cells (**b**) or *Lrp5/6* double KO and parental MDCK cells (**c**) were treated with exosome-depleted CS (input S100) from co-culture of GFP-Wnt3a/L cells with or without sFRP2-expressing cells. P100 (2nd) fractions from these cells were subjected to Western blotting. The amount of GFP-Wnt3a recovered in P100 pellets collected from CS of *Lrp5/6* double KO or parental MDCK cells was quantified with Image J and standardized against that from parental cells treated with exosome-depleted CS from co-culture of GFP-Wnt3a/L and control HEK293 cells (**d**). Data are shown as mean ± s.e. p < 0.05 is considered statistically significant. *p* value were calculated by two-way ANOVA followed by Turkey HSD test, n = 3 independent experiments.

Given that sFRP2 increases cell-surface binding of Wnt3a, sFRP2 is likely to enhance Wnt3a interactions with membrane proteins that maintain Wnt3a on the cell surface. Thus, we next examined effects of several membrane proteins on exosome-mediated re-secretion of Wnt3a utilizing Re-secretion assay #1. Since HSPG captures soluble ligands, including Wnt, on the cell surface, we investigated involvement of HSPG by expressing a membrane-tethered form of Heparinase III (HepIII) in secondary cells. The increase in exosome-mediated re-secretion of Wnt3a by sFRP2 was almost negated by HepIII in MDCK cells (Fig. 3a), indicating that glycan chains on HSPG are required for the sFRP2-dependent increase in exosomal re-secretion of Wnt3a.

We next investigated whether Wnt receptors (Frizzleds, Fzd) are involved in exosomal Wnt release under the influence of sFRP2. Mice and humans express 10 members of the Fzd family^48,49^. Since all *Fzd* genes had already been knocked out in HEK293 cells, we utilized this KO line (*Fzd1–10* KO)^50^ in our study (Supplementary Fig. 12). In *Fzd1–10* KO cells, exosomal release of GFP-Wnt3a was clearly reduced with or without sFRP2 (Fig. 3b), although a certain amount of GFP-Wnt3a was still released on exosomes.

To investigate whether Wnt/β-catenin signaling affects exosomal Wnt release, we asked whether LRP5 and LRP6, which act as co-receptors for Wnt3a on the cell membrane^51,52,53^ in activation of Wnt/β-catenin signaling, are required for the sFRP2-mediated increase in re-secretion of Wnt3a on exosomes. We generated *Lrp5* and *Lrp6* double knock-out (KO) MDCK cells using CRISPR/Cas9-mediated genome editing^54^ (Supplementary Fig. 13a) and examined their contributions to re-secretion of Wnt3a on exosomes. However, no significant change was detected in exosome-related Wnt3a release mediated by sFRP2 (Fig. 3c, d), although Wnt/β-catenin signaling was significantly reduced in double KO cells (Supplementary Fig. 13b). Thus, sFRP2 increases exosome-mediated Wnt re-secretion independently of Wnt/β-catenin signaling, or even if this signaling is involved, some mechanism that bypasses it may exist.

### sFRP2 increases HSPG binding and exosomal release of Wnt3a in embryos

**Fig. 4:**
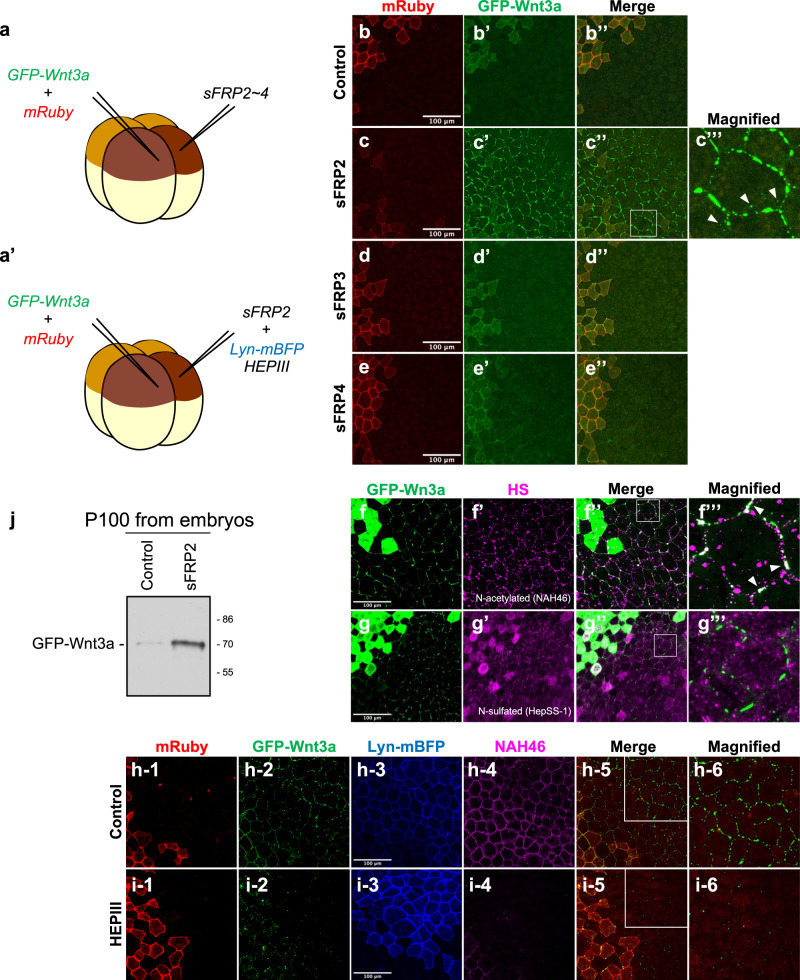
sFRP2 specifically increases Wnt3a attachment on cell surfaces in vivo. **a** Schematic figures of mRNA microinjection into fertilized *Xenopus* eggs. GFP-Wnt3a mRNA and various mRNAs, including sFRP mRNAs without (**a**) or with (**a’**) heparinase III (HEPIII) mRNA were injected into different blastomeres at the four-cell stage of *Xenopus* embryos. In most experiments, except that shown in (**j**), injected embryos were fixed with MEMFA at stage 11.5. GFP-Wnt3a-expressing cells are marked by membrane-bound Ruby (mRuby) expression (red), while neighboring cells expressing sFRP or controls are identified by Lyn-mBFP expression (blue). **b–e** Effect of sFRP proteins on the distribution of GFP-Wnt3a on surfaces of neighboring cells. Images of mRuby (**b–e**) and GFP-Wnt3a (**b’–e’**), as well as their merged images (**b”-e”**) are indicated. A magnified image of the area surrounded by a white line in (**c”**) is also shown (**c”’**). Injection with sFRP2 (**c**), but not with sFRP3 (**d**) or sFRP4 (**e**), increases GFP-Wnt3a accumulation on the cell surface and internalization into the cell (white arrowheads), compared to controls (**b**). **f**, **g** Correlation in the spatial pattern of GFP-Wnt3a and HSPG at the surface of sFRP2-expressing cells. Immunostaining of fixed embryos injected with GFP-Wnt3a and sFRP2, with antibodies specific for N-acetyl-rich (NAH46; **f**) or N-sulfo-rich (HepSS-1; **g**) HS chains. Images of GFP-Wnt3a (**f**, **g**) and HS chains (**f’**, **g’**), as well as their merged images (**f”**, **g”**), are indicated. A magnified image of the areas surrounded by white lines in (**f”** and **g”**) is also shown in (**f”’** and **g”’**). White arrowheads in (**f”’**) indicate co-localization of GFP-Wnt3a puncta and N-acetyl-rich HS puncta. **h**, **i** Effect of heparinase III (Hep III) on GFP-Wnt3a accumulation at the surface of sFRP2-expressing cells. Accumulation of GFP-Wnt3a on sFRP2-expressing cells was examined with (**i**) or without (**h**) expression of a membrane-tethered form of HepIII. Images of mRuby (**h-1**, **i-1**), GFP-Wnt3a (**h-2**, **h-2**), Lyn-mBFP (**h-3**, **i-3**), as well as merged images of mRuby and GFP-Wnt3a (**h-5**, **i-5**), are indicated. A magnified image of areas surrounded by white lines in (**h-5** and **i-5**) is also shown in (**h-6** and **i-6**). To confirm activity of HEPIII to remove the HS rich region, embryos were fixed and stained with NAH46 at stage 11.5 (**h-4**, **i-4**). Distribution of GFP-Wnt3a in sFRP2- expressing cells (**h-2**) was disrupted by expression of HEPIII (**i-2**). Scale bar; 100 μm. **j** Effect of sFRP2 in the exosome-mediated Wnt3a secretion in *Xenopus* embryos. Recovery of GFP-Wnt3a in the P100 fraction is shown. Equal numbers of embryos injected with GFP-Wnt3a with or without sFRP2 were dissociated at stage 11.5 and incubated another 5 h at 17 °C, followed by fractionation by ultracentrifugation at 100,000 × *g*. The P100 pellet was dissolved and subjected to Western blotting.

Previously, we showed that Wnt3a interacts with sFRP2 during diffusion in *Xenopus* embryos. In this case, sFRP2 increases Wnt3a localization on cell surfaces in embryos. To test whether this effect is specific for sFRP2, we injected GFP-Wnt3a mRNA and various sFRP mRNAs in different blastomeres at the four-cell stage and examined the effect of sFRP members on cell surface binding of GFP-Wnt3a at the gastrula stage (Fig. 4a). As observed in cell culture, sFRP2 specifically increased cell-surface binding of Wnt3a in embryonic tissue. Interestingly, sFRP1 showed a similar increase, but sFRP3 and sFRP4 did not (Fig. 4b–e, Supplementary Fig. 14). In addition, many GFP-Wnt3a puncta were detected inside sFRP2-expressing cells (Fig. 4c), indicating that endocytosis of GFP-Wnt3a was increased in the presence of sFRP2.

Since N-sulfated or N-acetylated HSPG proteins form separate clusters on cell membranes in *Xenopus* embryos^55^, we examined the association between GFP-Wnt3a puncta and these clusters. Interestingly, localization of GFP-Wnt3a puncta was apparently correlated with N-acetyl-rich HS puncta, but not with N-sulfo-rich HS puncta, which are reactive to NAH46 and HepSS-1 monoclonal antibodies, in the presence of sFRP2 (Fig. 4f–g). These GFP-Wnt3a puncta were actually dependent on HSPG, because they disappeared with expression of a membrane-bound form of HepIII^56^ (Fig. 4a’, h, i). We have already shown that N-sulfo-, but not N-acetyl-, HS puncta co-localize with Wnt signalosomes, where components involved in transduction of Wnt/β-catenin signaling are assembled across the cell membrane^55^. Therefore, the co-localization pattern of GFP-Wnt3a with N-acetyl-rich HS puncta supports the idea that sFRP2 increases cell-surface binding of Wnt3a independently of Wnt/β-catenin signaling.

To further examine involvement of exosomes in Wnt secretion from *Xenopus* embryonic cells, we injected GFP-Wnt3a and sFRP2 mRNAs into different blastomeres at the four-cell stage (Fig. 4a) and examined secreted Wnt3a on exosomes 24 h after injection (Fig. 4j). To recover secreted Wnt3a in blastocoel and the extracellular space in embryos, we dissociated *Xenopus* embryos into individual cells in modified phosphate buffer with EDTA, then incubated for 5 h, followed by ultracentrifugation 100,000 x *g*. Even in this situation, sFRP2 increased the amount of Wnt3a protein recovered in the P100 fraction. Taken together, these results show that interaction of sFRP2 with Wnt3a is specific not only in cell culture, but also in embryos.

### Wnt3a forms heterodimers with sFRP2, but not with other sFRPs

**Fig. 5:**
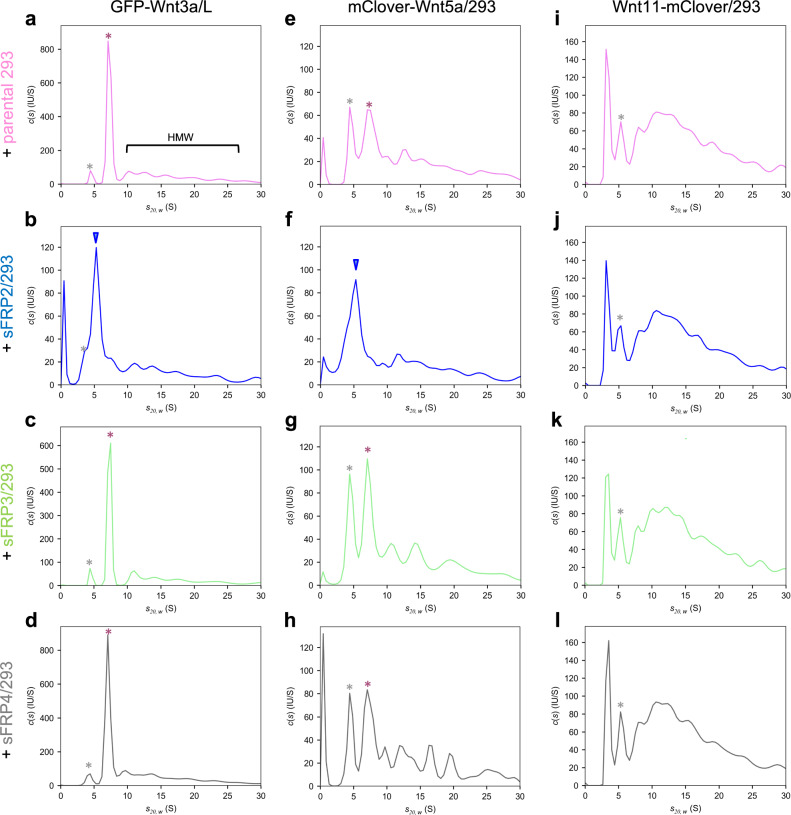
Analytical ultracentrifugation analyses of green fluorescent-tagged Wnt proteins with various sFRPs. AUC-FDS analysis of the CS of parental (pink; **a**, **e**, **i**), sFRP2- (blue; **b**, **f**, **g**), sFRP3- (green; **c**, **g**, **k**), or sFRP4-(gray; **d**, **h**, **l**) expressing HEK293 cells, co-cultured with GFP-Wnt3a/L (**a–d**), mClover-Wnt5a/HEK293 (**e–h**) or Wnt11-mClover/HEK293 (**i–l**). Peaks indicated by red asterisks correspond those of the Wnt/afamin complex (7.0 S peak, ~150 kD) while a bracket indicates high-molecular-weight (HMW) complexes. Since a small peak at ~4.2 S is detectable even in the CS of normal L cells, this fluorescence appears to be derived from serum components, probably albumin, associated with bilirubin (gray asterisks)^33^. In addition, a small peak at 3 S in the CS of Wnt11-mClover/HEK293 cells (**i–l**) appears due to degradation of Wnt11-mClover (Supplementary Fig. 15). As with GFP-Wnt3a, which forms a 1:1 complex with afamin, as previously reported (**a**), mClover-Wnt5a (**e**) also show peaks of the same size (S value), suggesting that mClover-Wnt5a forms a 1:1 complex with afamin. In the presence of sFRP2, these peaks disappear and a new peak (indicated by blue arrowheads) appears at 5.4 S, the size of which corresponds to Wnt/sFRP2 1:1 complex, in the CS of GFP-Wnt3a (**b**) or mClover-Wnt5a (**f**), but not Wnt11-mClover (**j**). Western blotting analysis shows that amounts of sFRP3 and sFRP4 in the CS were equal to or more than that of sFRP2 (Supplementary Fig. 15). Tagged Wnt5a and Wnt11 still retained their activities (Supplementary Fig. 16).

Wnt forms protein complexes with carrier proteins in the extracellular space. In vertebrates, several sFRP proteins act as carriers of Wnt in cell cultures and embryos. Since sFRP1 and sFRP2, but not sFRP3 or sFRP4, specifically increase cell-surface attachment of Wnt3a, we speculated that the former two and the latter two sFRPs may differ in their interactions with Wnt3a in the CS. To test this possibility, we directly examined whether sFRPs form complexes with Wnt3a in the CS. We investigated this using a non-invasive approach, i.e., analytical ultracentrifugation with a fluorescence detection system (AUC-FDS)^57–59^, which allowed us to directly measure the size of molecules of interest in CS without artificial manipulation using detergent. By AUC-FDS analysis, we previously showed that sFRP2 forms heterodimers with secreted GFP-Wnt3a in the CS of GFP-Wnt3a-expressing cells co-cultured with sFRP2-expressing cells^33^. Using this method, we tested interactions of Wnt3a with other sFRPs. Since the secretion level of sFRP1 was quite low for unknown reasons, we examined whether GFP-Wnt3a can form protein complexes with sFRP2, sFRP3 or sFRP4. In contrast to sFRP2, GFP-Wnt3a did not form heterodimers with sFRP3 or sFRP4 in the CS under the same conditions in which GFP-Wnt3a/sFRP2 heterodimers were detectable (Fig. 5a–d, Supplementary Fig. 15). Thus, only sFRP2 can form stable heterodimers with GFP-Wnt3a under conditions in which we examined the effect of sFRP proteins on Wnt3a in cell culture. Furthermore, when we compared GFP-Wnt3a/sFRP2 interaction with or without CS of cells expressing the extracellular domain of Frizzled8 (exFzd8), the peak corresponding to GFP-Wnt3a/sFRP2 heterodimerization was shifted to a peak corresponding to GFP-Wnt3a/exFzd8, showing that GFP-Wnt3a is more likely to associate with Fzd8 than sFRP2 (Supplementary Fig. 17). Based on these results, we speculated that formation of stable Wnt3a/sFRP2 heterodimers is involved in efficient attachment of Wnt3a to the cell surface, followed by increased endocytosis and exosome-mediated re-secretion of Wnt3a.

### Wnt specificity in the effect of sFRP2 on exosome-mediated re-secretion

**Fig. 6:**
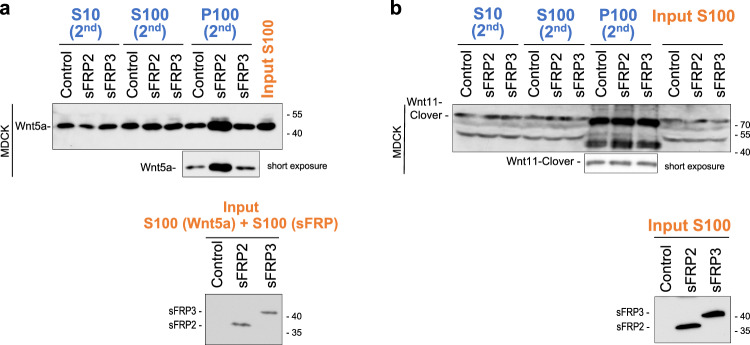
Re-secretion of exosomal Wnt5a, but not Wnt11, is increased specifically by sFRP2. Exosome-mediated re-secretion of Wnt5a and Wnt11 was examined according to the procedure shown in Supplementary Fig. 4a (Re-secretion assay #2) for (**a**), or Fig. 1a (Re-secretion assay #1) for (**b**). Western blot analysis of S10 (2nd), S100 (2nd), and P100 (2nd) for examining the specificity of sFRP to Wnt5a (**a**) and Wnt11 (**b**) proteins. S100 mixture from separately cultured CSs of non-tagged Wnt5a/L cells and sFRP2-, sFRP3-, expressing or control HEK293 were used to treat MDCK cells in (**a**). Exosome-depleted CS of Wnt11-mClover/HEK293 cells co-cultured with sFRP2-, sFRP3-expressing or control HEK293 cells were used to treat MDCK cells in (**b**).

We next examined whether sFRPs promote exosome-mediated re-secretion of other Wnt proteins, utilizing Re-secretion assay #1 or #2. Exosome-depleted CS (input S100 fraction) of Wnt5a-expressing cells mixed with that of sFRP2- or sFRP3-expressing cells were treated with MDCK or L cells. Wnt5a in lysate of secondary cells and re-secretion on exosomes was also promoted only by sFRP2 (Fig. 6a; Supplementary Fig. 18a, b). However, exosome-mediated re-secretion of Wnt11 and Wnt11 in lysate of secondary cells did not increase in the presence of sFRP2 (Fig. 6b, Supplementary Fig. 18c). On the other hand, AUC-FDS analysis indicated that mClover-tagged Wnt5a was able to form heterodimers with sFRP2, but not with sFRP3 or sFRP4 (Fig. 5e–h, Supplementary Fig. 16), as in the case of Wnt3a. But mClover-tagged Wnt11 did not form protein complexes with any of the three sFRPs (Fig. 5i–l, Supplementary Fig. 16). Thus, the ability to form heterodimers with sFRP is highly correlated with promotion of exosome-mediated re-secretion of Wnt proteins.

### Wnt heterodimerization increases re-secretion via exosomes

**Fig. 7:**
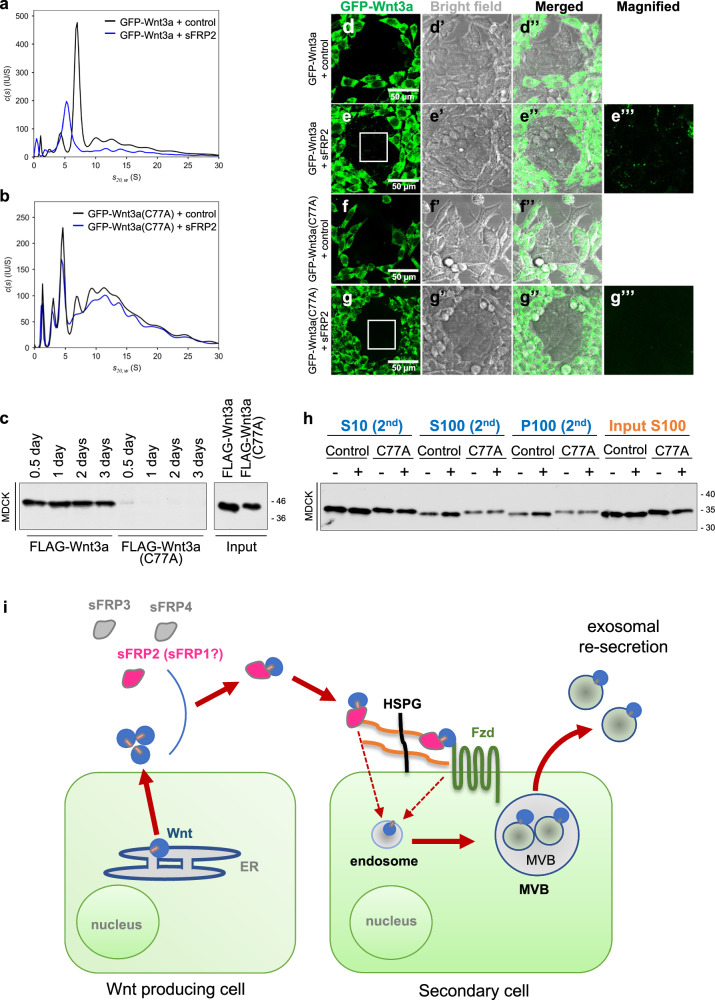
Wnt3a mutant defective in heterodimer formation with sFRP2 prevents re-secretion of Wnt3a on exosomes. Analysis with AUC-FDS of the CS from co-culture of GFP-Wnt3a (**a**) or GFP-Wnt3a (C77A) (**b**) expressing L cells with sFRP2-expressing or control HEK293 cells. In the presence of sFRP2, a shift in the peak position of control GFP-Wnt3a was observed, whereas peak positions of GFP-Wnt3a (C77A) remained largely unchanged. Thus, in contrast to control GFP-Wnt3a, heterodimers of GFP-Wnt3a (C77A) and sFRP2 were not formed. **c** Western blot for detection of FLAG-Wnt3a in lysate of MDCK cells treated with exosome-depleted CS of FLAG-Wnt3a or FLAG-Wnt3a (C77A) for indicated periods. **d–g** Distribution of GFP-Wnt3a (C77A) in sFRP2-expressing cells. To examine whether the Wnt3a (C77A) mutant is defective in distribution in the presence of sFRP2, GFP-Wnt3a- (**d**, **e**) or GFP-Wnt3a (C77A; **f**, **g**) producing L cells were co-cultured with sFRP2-expressing cells (**e**, **g**), or control HEK293 cells (**d**, **f**) from 2 days before observation. All images were processed by maximum intensity projection. Images of GFP-Wnt3a (**d–g**), bright field (**d’–g’**), and their merged images (**d”**–**g”**) are shown. Scale bar; 50 μm. Magnified images of areas surrounded by white lines in (**e**, **g**), are also shown in (**e”’**, **g”’**). **h** Examination of exosome-mediated secretion of Wnt3a (C77A) following Re-secretion assay #1. Western blot for detection of Wnt3a recovered in S10, S100 and P100 fractions collected from CS of MDCK cells cultured with S100 of non-tagged Wnt3a/L or FLAG-Wnt3a (C77A)/L co-cultured with sFRP2-expressing or control HEK293 cells are shown. **i** Exosomal Wnt re-secretion model.

If heterodimer formation and subsequent dissociation are crucial for endocytosis and exosome-mediated re-secretion of Wnt ligands, a mutant form of Wnt3a that cannot form heterodimers with sFRP2 should be defective in sFRP2-mediated enhancement of these processes. Previously, we found that a mutant form of Wnt3a, Wnt3a-C77A, in which Cys77 is substituted for Ala, aggregates, forming barely dissociable protein complexes^33,60^. In this study, using AUC-FDS analysis, we found that Wnt3a-C77A forms heterodimers poorly with sFRP2 (Fig. 7a, b). In addition, the amount of Wnt3a in the cell lysate was apparently decreased in MDCK cells co-cultured with CS of Wnt3a (C77A)-expressing L cells (Fig. 7c). Furthermore, we found that membrane association of GFP-Wnt3a-C77A (Fig. 7d–g) and exosome-mediated re-secretion of FLAG-Wnt3a-C77A (Fig. 7h) were not enhanced by sFRP2. Thus, heterodimer formation with sFRP2 seems to be crucial for sFRP2-mediated endocytosis and exosome-mediated re-secretion of Wnt3a (Fig. 7i).

## Discussion

In this study, we found that sFRP2 increased exosome re-secretion of several Wnt ligands in both cultured cells and *Xenopus* embryos (Fig. 7i). This effect of sFRP2 was canceled by expression of the membrane-bound form of heparinase. In addition, Wnt3a was co-localized with HSPGs in the presence of sFRP2 in embryos. These results suggest that sFRP2 increases Wnt association with HSPG on cell membranes, resulting in increased endocytosis and exosome-mediated re-secretion of Wnt. On the other hand, sFRP proteins were initially thought to act as antagonists, primarily by sequestering Wnt^61–63^. In addition to their antagonism of Wnt signaling, several sFRP proteins also act as carriers to transport Wnt ligands over long distances^32,64^. Therefore, these conflicting results, including results in this study, suggest that sFRP may have multiple roles in Wnt signaling and behavior in tissues.

One of the important points in understanding various functions of sFRPs is to characterize binding between Wnt and sFRPs. Wnt3a binds to four sFRPs (sFRP1, sFRP2, Frzb (sFRP3), sFRP4) with affinities in the nanomolar range^65^. However, since commercially available Wnt proteins are solubilized with high concentrations of detergents (1% CHAPS)^30^, previous studies were also conducted in the presence of detergent at the same concentration. Therefore, we believe that it is important to carefully consider the binding affinity between Wnt and sFRP under conditions that are more nearly physiological.

In this study, we measured Wnt3a and three sFRPs (sFRP2, sFRP3, sFRP4) secreted in culture supernatant, and examined whether they form stable complexes, utilizing AUC with a fluorescence detection system. Unfortunately, we have not yet been able to obtain CS containing concentrations of sFRP1 high enough for AUC analysis. Using this method, the size of the Wnt complex can be measured in culture supernatant because Wnt proteins are labeled with a fluorescent tag^33^. To our surprise, only sFRP2 and Wnt3a form stable heterodimers. Similarly, Wnt5a formed a stable heterodimer only with sFRP2, but Wnt11 could not form a complex with any of these three sFRPs. Since complex-forming ability is correlated with specificity of sFRP-induced cell surface accumulation and exosome-mediated re-secretion of Wnt proteins, we hypothesized that heterodimer formation under natural conditions is involved in accumulation of Wnt on cell membranes, which seems to be followed by exosome-mediated re-secretion. In fact, a Wnt3a mutant with impaired heterocomplex-forming ability^33^ apparently reduced cell membrane accumulation and exosome-mediated re-secretion. We believe that these results strongly support our hypothesis. Interestingly, several reports show that some sFRPs accumulate on HSPGs with N-acetyl-rich HS chains, suggesting that the affinity between sFRP and HSPG may also be another factor determining the binding of Wnt/sFRP heterodimers to HSPGs^55,66^. However, it is necessary to investigate in more detail whether increased binding of Wnt/sFRP2 heterodimers to HSPGs really contributes to increased Wnt endocytosis and exosomal re-secretion. At present, we cannot exclude the possibility that sFRP2 may also be involved in some other process of Wnt endocytosis, beside binding to HSPGs.

This study raises the question of how Wnt/sFRP2 heterodimers are incorporated into cells. Given that both cell surface binding and exosomal re-secretion of Wnts are dependent on HSPGs, it may be that Wnt/sFRP2 heterodimers are incorporated into cells with HSPGs. On the other hand, it also seems probable that Wnts, once trapped by HSPGs, are transported to other cell surface molecules that are incorporated into cells with Wnts. In terms of the latter possibility, this study shows that LRP5 and LRP6 of co-receptors^51,67,68^ are dispensable for the sFRP2-mediated increase of exosome-mediated re-secretion of Wnt ligands. This indicates that at least some molecular mechanism for signal transduction of Wnt ligands across the cell membrane is not required for Wnt endocytosis mediated by sFRP2. On the other hand, our results also indicate that some Fzd receptors, which are also be the molecules for the signal transduction, are involved in this endocytosis, because loss of all Fzds reduces exosome-mediated Wnt re-secretion. Since this reduction was not complete, it is probable that both Fzd-dependent and -independent mechanisms are involved in Wnt endocytosis enhanced by sFRP2. In the former case, some Wnts bound to HSPGs may be transferred to Fzd and incorporated into the cell, because our AUC result shows that exFzd8 associates more readily with GFP-Wnt3a than FRP2. It will be interesting to learn how differences in endocytic mechanisms affect Wnt signaling and re-secretion.

Interestingly, three members of the sFRP family proteins, sFRP1, sFRP2, and sFRP5 are more similar in amino acid sequence than sFRP3 and sFRP4. Thus, we assume that some structural characteristics common to the former group may be involved in binding Wnt3a and Wnt5a, which results in increased cell surface binding and exosomal re-secretion. In this study, we showed that sFRP1 increases cell surface accumulation and exosomal re-secretion of Wnt3a. Although AUC analysis with sFRP1 has not yet succeeded, due to failure to establish a cell line that secretes high amounts of sFRP1, our results suggest that some structural characteristics common to the sFRP2-related subfamily may be crucial for binding to Wnt proteins.

Given that sFRP2 forms a stable complex with Wnt3a and Wnt5a, results of this study indicate that sFRP2 is directly involved as a carrier protein in extracellular transport of these Wnt proteins and indirectly promotes their re-secretion on exosomes. Which mode of Wnt transport is more efficient in dispersing Wnt proteins over long distances is still uncertain. Since it is assumed that each mode of Wnt transport has its own characteristics, the extent to which these two modes are used can affect the spatial pattern of Wnt activation in tissues. As far as we have investigated in cell culture, the amount of Wnt protein secreted on exosomes is much less than that which directly forms heterodimers with sFRP2 in the CS. Therefore, we speculate that carrier-protein-mediated transport may be the dominant means of transport of Wnt proteins and that exosome-mediated transport may serve a complementary function, at least in conditions similar to our cell culture system. On the other hand, it seems plausible that exosome-mediated re-secretion is involved in fine tuning the spatial pattern of Wnt signaling in tissues. In many developing tissues, sFRPs are expressed with Wnt and are likely to modify Wnt gradients. In cases in which sFRP2 acts as an antagonist to Wnt ligands, sFRP2-mediated exosomal re-secretion should change the impact of sFRP2 on Wnt signaling from negative to positive. As a result, the extent of this change should affect the spatial pattern of Wnt signaling gradients. Since the range of exosome-mediated Wnt transfer is still controversial, it is uncertain how long exosomal resecretion affects Wnt gradient formation. Whether and how heterodimer-formation specificity of sFRP-mediated exosomal resecretion is involved in regulation of Wnt signaling will have to be clarified in future studies.

## Methods

### Cell culture and transfections

L and HEK293 cells were kindly provided by Dr. Masatoshi Takeichi (RIKEN), and the MDCK-II cell line was kindly provided by Dr. Tetsuhisa Otani (NIPS). *Fzd 1–10* KO HEK293 cells were were a generous gift from Dr. Benoit Vanhollebeke (UNI, Belgium)^50^, and SuperTopFlash/HEK293 cells were kindly provided by Dr. Tadasuke Tsukiyama (Hokkaido Univ)^69^.

MDCK, L and HEK293 cells were cultured in DMEM or DMEM:Ham’s F12 (1:1) medium supplemented with 8.3% FBS and antibiotics. L cells stably expressing FLAG-GFP-tagged mouse Wnt3a, FLAG-GFP-tagged mutant Wnt3a (GFP-Wnt3a(C77A)), FLAG-tagged mouse Wnt3a, FLAG-tagged mutant Wnt3a (Wnt3a(C77A)), non-tagged Wnt3a, non-tagged Wnt5a, and HEK293 cells stably expressing mouse sFRP2-FLAG, or mouse Fzd8-CRD-MycHis (exFzd8) were established previously^33,70^ and maintained in culture containing 400 µg/mL G418 or 4 µg/mL Blasticidin S. L cells stably expressing FLAG-mCherry-tagged mouse Wnt3a under the control of the PGK promoter, HEK293 cells stably expressing FLAG-mClover-mouse Wnt5a and mouse sFRP1-FLAG and sFRP3-FLAG under control of the CMV promoter, mouse Wnt11-mClover under control of the PGK promoter, and mouse sFRP4-FLAG under control of the Tet-on promoter, were established by transfection of plasmid constructs with GeneJuice transfection reagent (Millipore), or the calcium phosphate method, followed by drug screening with 400 µg/mL G418, 1200 µg/mL G418, or 6 μg/mL Blasticidin S and by Western blotting after picking colonies.

HEK293 cells stably expressing both sFRP2-FLAG and pHluorin-M153R-CD63-mTagBFP were established by transfection of plasmid constructs with GeneJuice transfection reagent into sFRP2 producing HEK293 cells, followed by drug screening with 1000 µg/mL G418 and picking colonies. pHluorin-M153R-CD63-mTagBFP was generated by substituted mScarlet to mTagBFP from original published construct (Addgene #172118), then cloned into pCS2 neo.

HEK293 cells stably expressing HepIII-HA-GPI was established by transfection of plasmid construct with GeneJuice transfection reagent, followed by drug screening with 1000 µg/mL G418 and picking colonies and Western blotting with anti HA, rat monoclonal antibody.

### Antibodies and Western blotting

To examine protein levels in samples prepared in this study, Western blotting was carried out by following a standard protocol. Briefly, samples were diluted in 2x SDS-PAGE sample buffer composed of 4% SDS, 20% glycerol, 0.1 M Tris-HCl (pH6.8), 9% 2-mercaptoethanol, and 0.01% bromophenol blue, heated at 95 ^o^C for 10 min and loaded on 10%, or 12% acrylamide gels for electrophoresis. Proteins were transferred to a PVDF membrane and blocked in 5% skim milk, before being incubated with primary antibodies (Supplementary Table 1).

### Preparation of exosomes

We report this procedure basically according to the Minimal Information for Studies of Extracellular Vesicles 2018 guidelines^71^. To prepare exosome-depleted CS, mouse L cells stably expressing FLAG-GFP-tagged, FLAG-tagged or non-tagged Wnt3a were cultured with parental HEK293 cells or HEK293 cells stably expressing sFRP2-FLAG, sFRP3-FLAG, sFRP4-FLAG, or exFzd8-MycHis at a 9:1 ratio and an initial density of 2 ×10^6^ cells on 100-mm-culture plates for 2 days. Then, medium was removed, and cells were rinsed twice with PBS (-) or DMEM containing 4% exosome-free FBS and incubated with fresh DMEM containing 8% exosome-free FBS for 2 more days. The CS was then collected and processed by centrifugation.

To examine the effect of sFRP2 on re-secretion of Wnt3a on exosomes, cells were treated with exosome-depleted CS from GFP-Wnt3a/L cells co-cultured with parental HEK293 cells or HEK293 cells expressing sFRP1, sFRP2, sFRP3, or exFzd8 (Fig. 1a). Mouse L cells stably expressing FLAG-GFP-tagged, FLAG-tagged or non-tagged Wnt3a were cultured with parental HEK293 cells or HEK293 cells stably expressing sFRP1-FLAG, sFRP2-FLAG, sFRP3-FLAG, or exFzd8-MycHis at a 9:1 ratio and an initial density of 2 ×10^6^ cells on 100-mm-culture plates for 2 days. Then, medium was removed, and cells were rinsed twice with PBS (-) or DMEM containing 4% exosome-free FBS and incubated with fresh DMEM containing 8% exosome-free FBS for 2 more days. The CS was then collected and exosome-depleted CS from these cultures was prepared by the following procedure^72^. The CS was first centrifuged at 1500 × *g* for 10 min, then 10,000 × *g* for 30 min to remove cellular debris, and supernatant was recovered by further centrifugation at 100,000 × *g* for 18 h at 4 ^o^C with Beckman Type 45Ti rotor. This was used as exosome-depleted CS (Fig. 1a). Then exosome-depleted CS was added to the culture of secondary cells, including MDCK, L, and HEK293 cells. We refer to this as Re-secretion assay #1.

Alternatively, secondary cells were treated with exosome-depleted CS (input S100 fraction) from GFP-Wnt3a/L mixed with that from sFRP2-, sFRP3-, sFRP4-, exFzd8-expressing or control HEK293 cells in a 4:1 ratio (Supplementary Fig. 4a). We refer to this as Re-secretion assay #2. In both Re-secretion assay #1 and #2, the CS of secondary cells was collected 24 h after treatment, and processed using the following ultracentrifugation: 1500 × *g* for 10 min, 10,000 × *g* for 30 min (the supernatant was referred to as S10 (2nd)), 100,000 × *g* for 90 min at 4 ^o^C with Beckman Type 45Ti rotor, TLA-100.2, or TLA-100.3 rotor (recovered supernatant was referred to as S100 (2nd) and the exosome-containing pellet was termed P100 (2nd)).

To examine the possibility that sFRP2 enhances direct incorporation of GFP-Wnt3a into exosomes independently of cells, GFP-Wnt3a incorporation into exosomes was examined in cell-free conditions, in which, exosome-depleted CS (input S100 fraction) of GFP-Wnt3a/L and that of sFRP2-expressing HEK293 or control HEK293 were mixed with S10 of parental MDCK cells, from which exosomes had not yet been removed. These were further incubated at 37 ^o^C for 24 h. Then, mixtures were centrifuged to obtain the P100 fraction (Supplementary Fig. 7).

### *Xenopus* microinjection

This study was performed in accordance with Guidelines for Animal Experimentation of the National Institutes of Natural Sciences, with approval of the Animal Care and Use Committee (IACAC) of the National Institutes of Natural Sciences. Microinjection experiments in *Xenopus laevis* eggs were carried out according to standard methods^32,73^, as follows. Eggs for fertilization were obtained from gonadotropin (ASKA Pharmaceutical) injected female frogs. Artificial fertilization was done using testis homogenate, followed by de-jellying using 4% L-cysteine (pH 7.8) and incubated at 17°C in 0.1x Steinberg’s solution. mRNAs synthesized with an mMESSAGE mMACHINE^TM^ SP6 Transcription SP6 kit (Invitrogen) were microinjected into the animal caps of ventral blastomeres at the 4-cell stage and observed at the gastrula stage. mRNAs were injected at a final concentration as follows: *FLAG-GFP-mWnt3a*; 500 pg/embryo, *mRuby2-KRas*; 100 pg/embryo, *sFRP1*, *sFRP2*, *sFRP3*, or *sFRP4*; 1000 pg/embryo, *Lyn-mTagBFP2*; 100 pg/embryo, *HepIII-HA-GPI* (membrane-tethered HepIII); 400 pg/embryo.

To collect exosomes secreted from *Xenopus* embryos, 30 embryos injected with *FLAG-GFP-Wnt3a* together with or without *sFRP2* were dissociated in 1.2 mL of modified phosphate buffer (50 mM Na Phosphate, 35 mM NaCl, 1 mM KCl) with EDTA at stage 11.5. This 1.2 mL of embryo-dissociated buffer was then incubated for 5 h at 17°C and followed by the exosome preparation protocol to obtain the P100 fraction, as described above.

For immunostaining, microinjected embryos were treated using our standard protocol^55^. Fixed embryos were incubated with HepSS-1 (mouse monoclonal antibody, IgM; prepared in-house) or NAH46 (mouse monoclonal antibody, IgM; prepared in-house) in TBST overnight at 4 °C, washed 3 times with TBS, and incubated with Alexa Fluor 647-conjugated secondary antibody.

### Chemical inhibitor

MDCK cells grown to 80% confluency were treated with S100 of GFP-Wnt3a with or without sFRP2 and 10 µM GW4869 (Sigma-Aldrich D1629) or DMSO for 24 h before preparation of exosome fractions.

### Tracking of mCherry-Wnt3a in receiving cells

The dual-fluorescence reporter allows us to track cytosolic CD63-positive structures with pH-insensitive blue fluorescence under acidic condition, especially MVB. Dual blue and pH-sensitive green fluorescence can be detected when MVB fuses to plasma membrane and exosomes are released into neutral environment. To monitor Wnt3a movement in neighboring cells, we took advantage of this novel construct to track mCherry-Wnt3a localization during MVB formation, fusion and exosome secretion. L cells stably expressing mCherry-Wnt3a were co-cultured with HEK293 cells stably producing sFRP2 and pHluorin-M153R-CD63-mTagBFP on glass bottomed plates at a ratio of 3:2 for 1 day before observation with confocal microscopy. To inhibit MVB formation, 12 h after culture, cells were treated with DMEM medium containing 10 µM GW4869 or DMSO. Observation was done 24 h further after chemical inhibitor treatment.

### Analytical ultracentrifugation

All analyses conducted by analytical ultracentrifugation with a fluorescence detection system (AUC-FDS) were carried out according to the procedure shown below^33^. CSs for AUC-FDS analysis was prepared with FluoroBrite DMEM (Gibco) with 8% FBS from either GFP-Wnt3a/L, GFP-Wnt3a (C77A)/L, mClover-Wnt5a/HEK293, or Wnt11-mClover/HEK293 cells with sFRP2-, sFRP3-, or sFRP4-expressing or parental HEK293 cells. CSs were collected from confluent cultures, centrifuged at 1500 × *g* for 10 min, 10,000 × *g* for 30 min to remove cell debris, and then subjected to analytical ultracentrifugation (AUC; Beckman Coulter) with a fluorescence detection system (FDS; AVIV Biomedical). All AUC experiments were conducted using Beckman 12-mm charcoal-filled, Epon, double-sector centerpieces at 20 °C and at a rotor speed of 42,000 rpm. A *c*(*s*) model of SEDFIT were used for analysis of acquired data^58^. Resulting sedimentation coefficient distributions were transformed to standard conditions of water at 20 °C by considering density and viscosity. Density was measured using a density meter (Anton Paar DMA4500), and viscosity of FluoroBrite DMEM with 8% FBS was measured with a Lovis 2000ME viscometer.

### Wnt internalization

Cells at 80% confluency were treated with exosome-depleted CS collected from co-culture or mixture of those from individual cultures stably expressing cell lines for indicated periods at 37 °C in CO_2_ incubator, prior to being washed twice with PBS (-), before being lysed with SDS-PAGE sample buffer. For assays performed at 4 °C, cells and exosome-depleted CS were pre-cooled on ice for 10 min before treatment, kept at 4°C, and then collected after the indicated time point.

### Genome editing with the CRISPR/Cas9 system

*Lrp5/6* dKO cells were generated in MDCK cells by electroporation with Cas9-gRNA RNP complexes^54,67,68^. For *Lrp5* targeting, gRNA1 - 5’ GATGAAGCTGAGCTTGGCAT 3’, gRNA2 - 5’ GCTGAGCACTTGAATATCCA 3’, gRNA3 - 5’ GGTCAAGGTCCTGCCAGAAG 3’ were used. For *Lrp6* targeting, gRNA1 - 5’GAGAATGCTACAATTGTAGT 3’, gRNA2 – 5’ GTGGACTTTGTGTTTGGTCA 3’, gRNA3 – 5’ GGATCTAAGGCAATAGCTCT 3’ were used. Potential target cells were screened by Western blotting using LRP5 and LRP6 antibody, and finally confirmed by Sanger sequencing of genomic PCR products.

### Wnt3a activity assay

To monitor activity of Wnt3a in *Lrp5*-KO, *Lrp6*-KO, or *Lrp5/6* dKO MDCK cells and *Fzd1–10* KO HEK293 cells, we transfected pre-plated cells with plasmids expressing firefly luciferase, which is inducible under control of a promoter containing 8 tandem repeats of the TCF/LEF1 binding sites (SuperTopFlash reporter)^33,74,75^, and a *Renilla* luciferase-expressing vector under control of the TK promoter, which served as a transfection internal control. Lipofectamine LTX Plus (Thermo Fisher Scientific) was used to transfect MDCK cells and GeneJuice (Millipore) was used to transfected HEK293 cells. At 12 h post-transfection, culture medium was changed to conditioned medium of Wnt3a/L or control cells. Cells were harvested after another 12 h and luciferase activity was monitored using a Dual-Luciferase® Reporter Assay System (Promega).

Activity of fluorescently tagged Wnt5a and Wnt11 was monitored by examining activity antagonistic to Wnt3a/β-catenin signaling. A mixture of CSs of Wnt3a expressing L cells and those of parental HEK293, or HEK293 cells expressing Wnt5a or Wnt11 was used to treat SuperTopFlash (STF)/HEK293 cells that stably contain the firefly luciferase reporter gene under control of a promoter with 8 tandem repeats of TCF/LEF1 binding sites^69^. Twelve hours after treatment, cell lysates were harvested and luciferase activity was monitored using a Luciferase Assay System (Promega).

Wnt/β-catenin signaling activity of exosome fractions was monitored by adding the P100 exosome fractions to STF/HEK293 cells. After incubation for 24 h, cell lysates were collected and firefly luciferase activity was monitored as described above.

### Microscopy and image processing

Cultured cells and embryos were observed with a Leica confocal microscope (SP8) with 40x objective and images were processed using ImageJ. To examine the effect of sFRP on Wnt3a distribution, 2 × 10^5^ cells of FLAG-GFP-Wnt3a/L cells and sFRPs-FLAG/HEK293 cells were cultured in a ratio 3:2 for 2 days on glass-bottomed dishes pre-treated with a collagen mixture (Cellmatrix). Cells were fixed with MEMFA for 10 min, and then washed 3x with TBS. For tracking of GFP-Wnt3a internalization in the presence of sFRP2, living cells were treated with FM 4–64 FX (Invitrogen: F34653) at a final concentration of 5 µg/mL and monitored by time course imaging.

### Statistics and reproducibility

Error bars indicate s.e. Statistical significance was calculated by one-way ANOVA, two-way ANOVA followed by Turkey HSD test or Student’s t-test where appropriate and described in figure legends, using R and Excel softwares. Differences were considered statistically significant at p ≤ 0.05. All quantitated results were analyzed from at least three independent experiments.

### Reporting summary

Further information on research design is available in the Nature Portfolio Reporting Summary linked to this article.

### Supplementary information

Supplementary Information Figures

Description of Additional Supplementary Files

Supplementary Data

Supplementary Movie 1

Supplementary Movie 2

Supplementary Movie 3

Reporting Summary

## Data Availability

Raw data associated with figures are included in a Supplementary Data file. All other data are available in the article and Supplementary Information, including full blot images, which are provided in Supplementary Fig. 19.
