# Supplementary material for: Soluble Frizzled-related proteins promote exosome-mediated Wnt re-secretion
Source: Commun Biol. 2024 Mar 1;7:254. doi: 10.1038/s42003-024-05881-8 (PMC10907715; doi:10.1038/s42003-024-05881-8)
Supplement: Supplementary file 1 — Supplementary Information Figures [file 42003_2024_5881_MOESM1_ESM.pdf]

Supplementary Information

Soluble Frizzled-related proteins promote exosome-mediated Wnt re-secretion

Thi Hong Nguyen Tran, Ritsuko Takada, Elena Krayukhina, Takahiro Maruno, Yusuke Mii, Susumu Uchiyama, and Shinji Takada [*]

[*]Corresponding author: stakada@nibb.ac.jp

This PDF file includes: Supplementary Figures 1-19,
Legends of Supplementary Movies 1, 2 and 3,
Supplementary Table 1

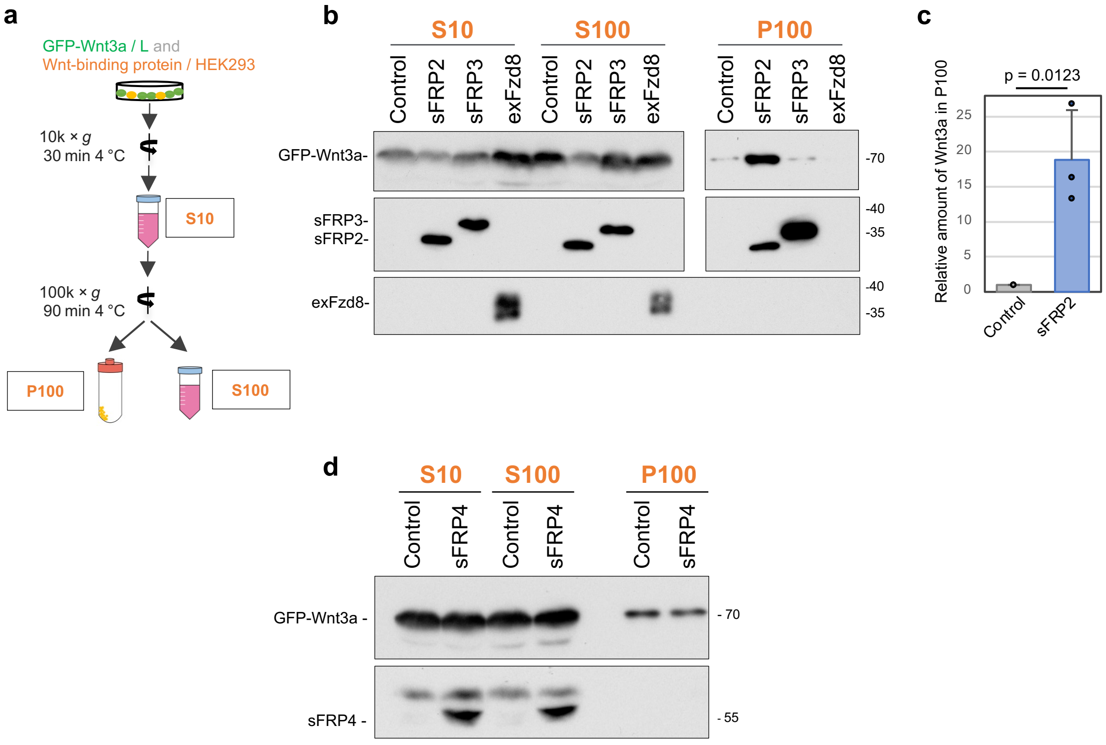

Supplementary Figure 1: Effect of different Wnt-binding proteins on exosome-mediated secretion of GFP-Wnt3a

a-d. Exosome-mediated secretion of GFP-Wnt3a into the co-culture supernatant of GFP-Wnt3a- expressing L (GFP-Wnt3a/L) cells with sFRP2-, sFRP3-, sFRP4 and exFzd8-expressing or control HEK293 cells. The experimental procedure is shown in **a.** By ultracentrifugation of culture supernatant (CS) of the co-culture at 100,000 x *g* for 90 min, exosome-depleted (S100) and the exosome-enriched (P100) fractions were collected. The amount of GFP-Wnt3a, sFRP2, sFRP3, sFRP4 and exFzd8 in each fraction, as well as input CS (S10), were examined by Western blotting (**b, d**). Each membrane was blotted separately in **b, d**.

Relative amounts of GFP-Wnt3a recovered in P100 fractions from co-culture supernatant of GFP-Wnt3a/L with sFRP2-expressing or control HEK293 cells shown in (**b)** were quantified using Image J software and results are shown as mean ± s.e (**c**). $p < 0.05$ was considered statistically significant, using two-sided Student's t tests, n = 3 independent experiments.

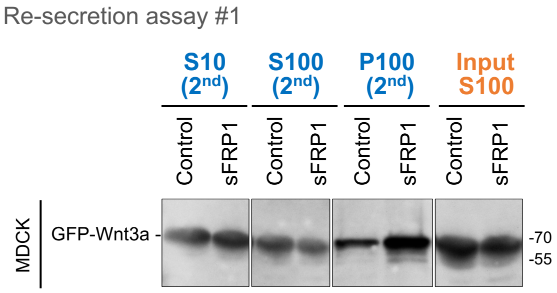

Supplementary Figure 2: Increase of exosome-mediated secretion of GFP-Wnt3a by sFRP1 in MDCK cells. Experiments shown in Figure 1a were performed with MDCK cells cultured with exosome-depleted co-cultured CS (input S100) from GFP-Wnt3a-producing L cells with sFRP1-FLAG producing or control HEK293 cells (by Re-secretion assay #1).

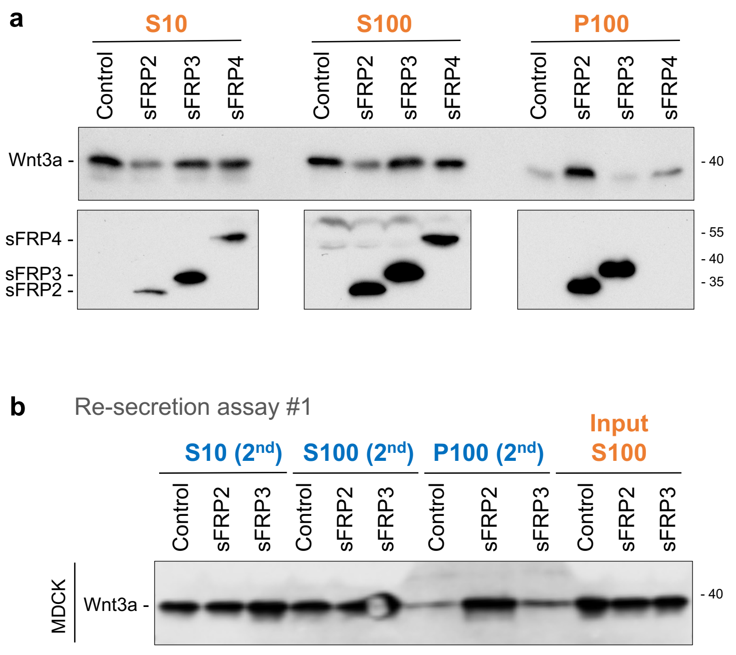

Supplementary Figure 3: Increase of exosome-mediated secretion of non-tagged Wnt3a by sFRP2 in L and MDCK cells.

Experiments shown in Supplementary Figure 1a and Figure 1a were performed by replacing GFP-Wnt3a-expressing L cells with non-tagged Wnt3a-expressing L cells. Western blot analysis to detect the amount of Wnt3a recovered in the S100 and P100 fractions from the CS of non-tagged Wnt3a-producing L cells co-cultured with sFRP-producing or control HEK293 cells (by experimental procedure in Supplementary Figure 1a; **a**) or MDCK cells cultured with exosome-depleted co-cultured CS (input S100) from non-tagged Wnt3a-producing L cells with sFRP-producing or control HEK293 cells (by Re-secretion assay #1; **b**). Each membrane was blotted separately in **a**.

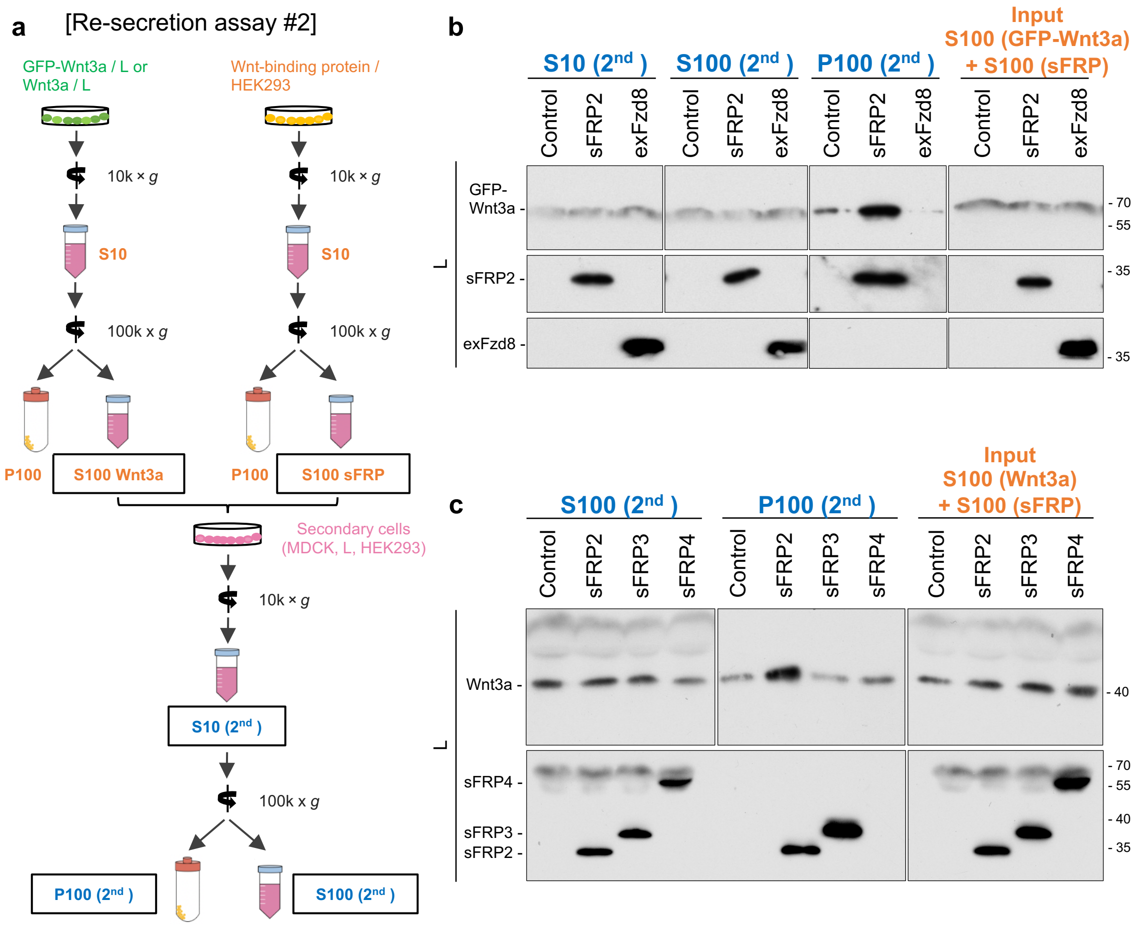

Supplementary Figure 4: Increased exosome-mediated secretion of GFP-Wnt3a with separately prepared sFRP2.

a. Schematic figure represents the experimental procedure using Re-secretion assay #2. Separately prepared exosome-depleted CSs of Wnt3a (S100) and of sFRP-, exFzd8-expressing, or control HEK293 cells were mixed and applied to secondary cells. After 24 h incubation at 37ºC, CSs were collected.

b. Western blot analysis examining amounts of GFP-Wnt3a from L cells treated with a mixture of S100 GFP-Wnt3a and S100 sFRP2, exFzd8, or control in a 4:1 ratio.

c. Western blot analysis examined amounts of non-tagged Wnt3a from L cells treated with a mixture of S100 GFP-Wnt3a and S100 sFRP2, sFRP3, sFRP4, or control in a 1:9 ratio.

Each membrane was blotted separately in **b, c**.

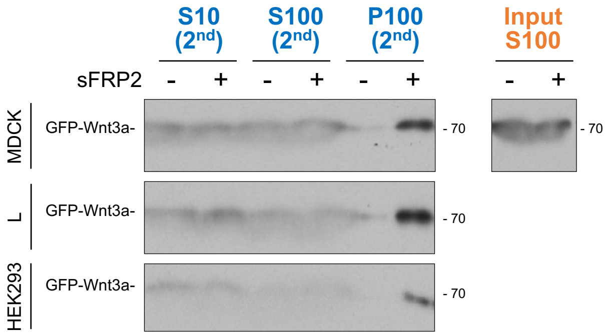

Supplementary Figure 5: Increase of exosome-mediated secretion of GFP-Wnt3a by sFRP2 in various cell lines.

Experiments shown in Figure 1a (Re-secretion assay #1) were carried out utilizing various cell lines. MDCK, L, and HEK293 cells were treated with exosome-depleted co-cultured CSs (input S100) from GFP-Wnt3a-producing L cells and sFRP2-producing or control HEK293 cells, followed by P100 (P100 (2nd)) fractionation.

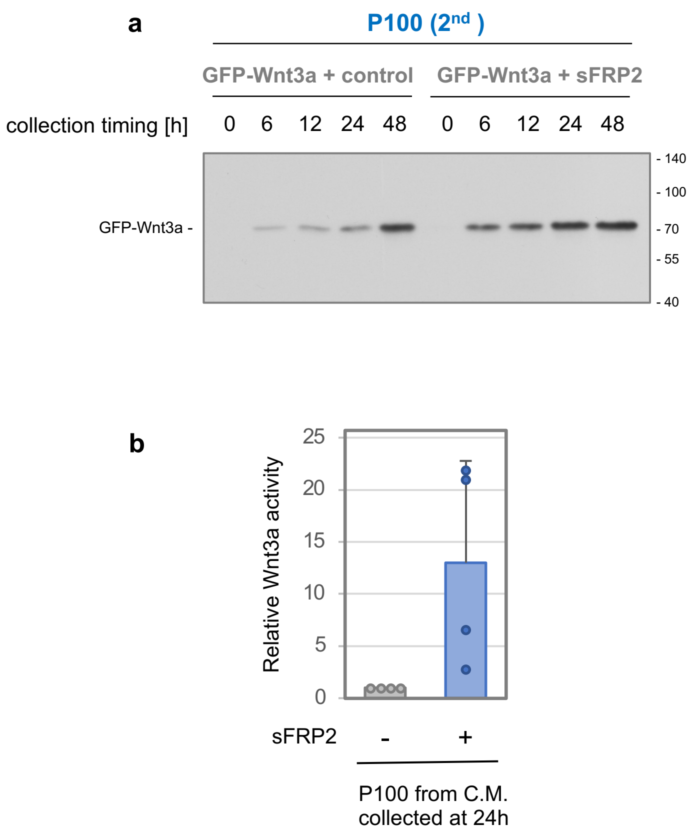

Supplementary Figure 6: Time-dependent re-secretion of exosomes from L cells and an increase of Wnt activity in P100 (exosome fractions)

a. Time course of GFP-Wnt3a amounts in exosome fractions, P100, prepared after treatment of exosome-depleted CS of GFP-Wnt3a expressing L cells preincubated with CS of sFRP2-expressing or control HEK293 cells, as shown in Re-secretion assay #2.

b. Wnt activity was examined 24 hr after treatment of the exosome fractions (2nd P100) with SuperTopFlash/293 cells, which stably contain a Wnt-reporter gene, firefly-Luciferase gene is inducible under the control of a promoter containing 8 tandem repeats of the Tcf binding sites. Relative activities to sFRP(-) condition in 4 different experiments are indicated. Exosome fractions were prepared as shown in **a**. $p < 0.05$ was considered statistically significant, using two-sided Student's tests.

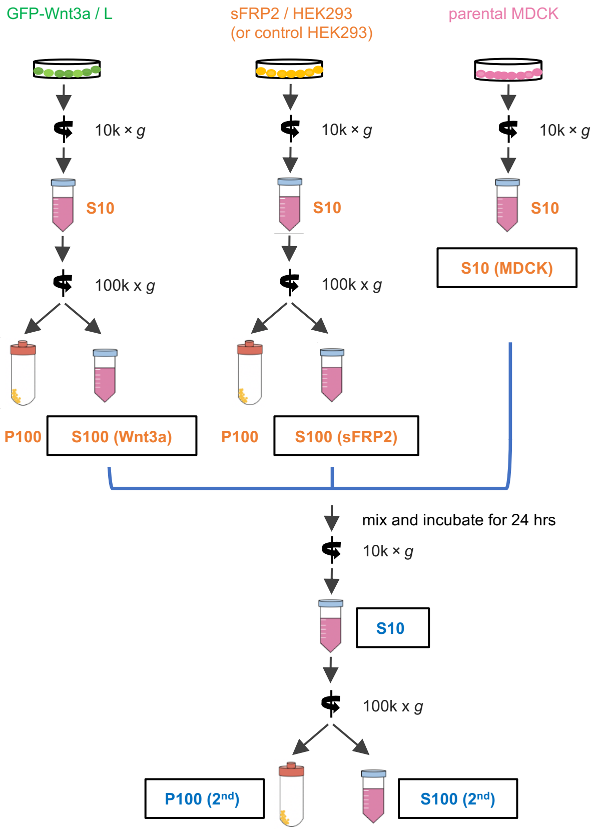

Supplementary Figure 7: Exosome-mediated secretion of GFP-Wnt3a in an incubation without cell treatment.

The experimental procedure of Figure 1f. Separately prepared exosome-depleted CSs (S100) of GFP-Wnt3a/L and that of sFRP2-expressing (or control) HEK293 cells were mixed with S10 of parental MDCK cells, which still maintains secreted exosomes. This mixture was incubated in tubes at 37ºC for 24 h, followed by P100 fractionation.

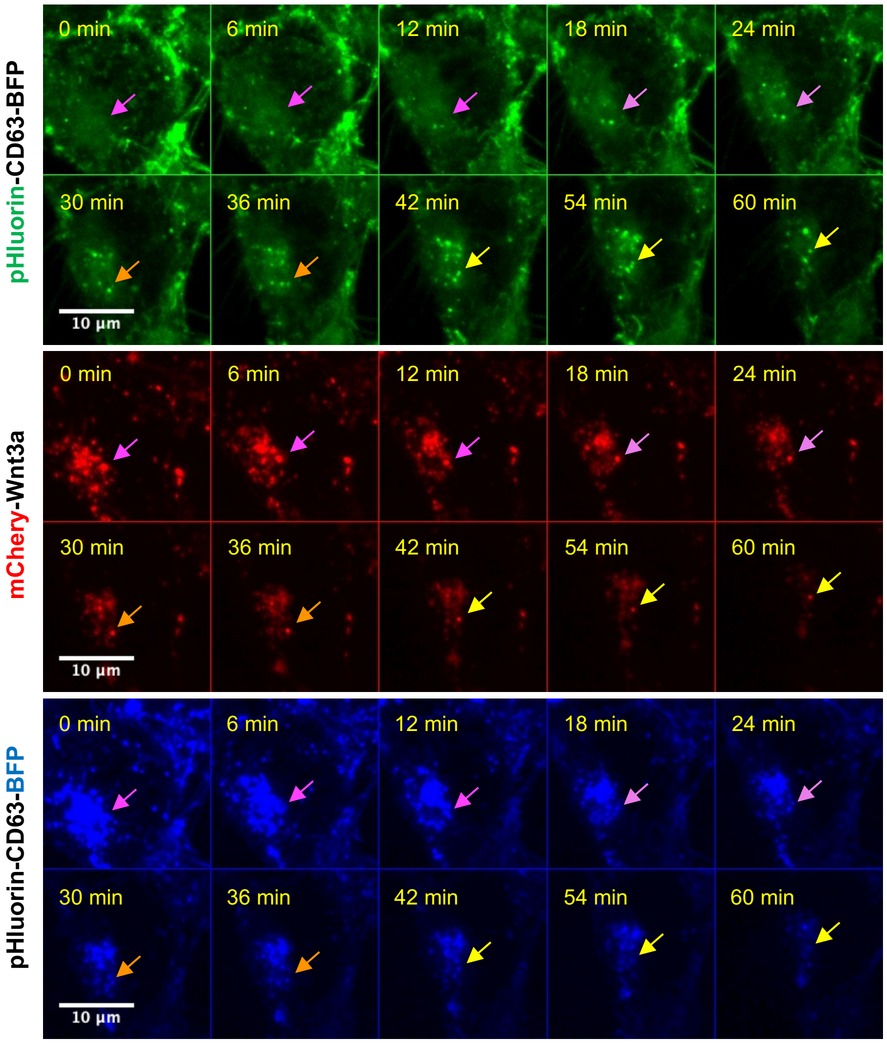

Supplementary Figure 8: Distribution of intracellular, extracellular CD63 and mCherry-Wnt3a in sFRP2-expressing cells.

This figure is supplementary to Figure 1x. It shows original images taken with three fluorescence channels. GFP channel indicates extracellular CD63, while BPF one indicates intracellular CD63.

BFP signal clearly overlaps the mCherry signal at all time points, while the GFP signal gradually accumulates and overlaps with BFP and mCherry puncta from time point 18 min, indicating that Wnt3a is initially colocalized with the endosomes (0 min to 18 min). Then it was released and colocalized with extracellular CD63 vesicles. Scale bar; 10 μm.

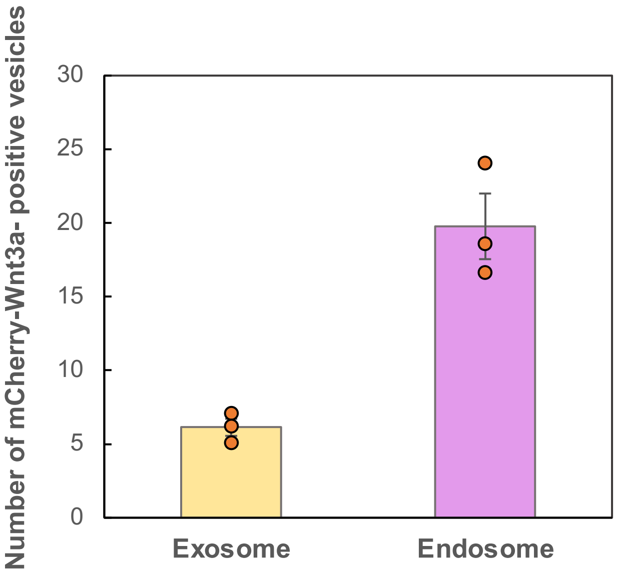

Supplementary Figure 9:

This is supplementary for Figure 1h-x.

Quantification shows the number of mCherry-Wnt3a-posititive vesicles normalized by the number of cells after co-culture of mCherry-Wnt3a/L and pHluorin-CD63-mTagBFP co-expressing sFRP2/HEK293 cells. Puncta double-positive for mCherry and mTagBFP indicate mCherry-Wnt3a-containing endosomes (magenta column) while those triple-positive for mCherry, GFP, and mTagBFP indicate mCherry-Wnt3a-containing exosomes (yellow column).

Data are expressed as means ± s.e, in 3 different images with 12, 17 and 12 cells, processed by maximum intensity projection.

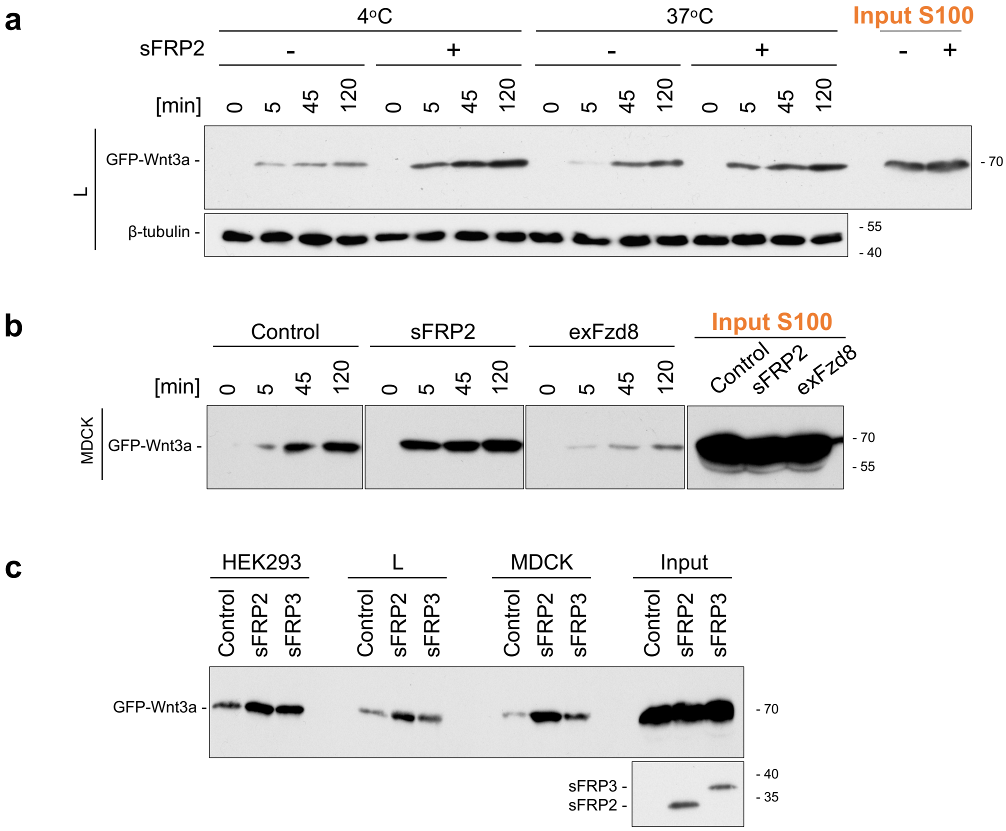

Supplementary Figure 10: GFP-Wnt3a in cell lysates is increased by sFRP2

a. Time course of GFP-Wnt3a in L cell lysates prepared at indicated times after treatment of exosome-depleted CS (S100) from GFP-Wnt3a-expressing L cells and separately prepared S100 from sFRP2-expressing or control HEK293 cells (by Re-secretion assay #2). Experiments were performed at 4 ºC and 37 ºC. Amounts of GFP-Wnt3a in L-cell lysates were examined after treatment with exosome-depleted CS with or without sFRP2. As with MDCK cells, GFP-Wnt3a amounts increased not only at 37ºC, but also at 4 ºC by sFRP2.

b. Time course of GFP-Wnt3a amounts in MDCK cell lysates prepared after treatment of exosome-depleted CS (input S100) of GFP-Wnt3a-expressing L cells co-cultured with sFRP2- or exFzd8-expressing or control HEK293 cells (by Re-secretion assay #1).

c. The amount of GFP-Wnt3a in HEK293, L, and MDCK cells prepared 2 h after treatment of exosome-depleted CS of GFP-Wnt3a-expressing L cells co-cultured with sFRP2- or sFRP3-expressing or control HEK293 cells at 37ºC. Note that as in MDCK cells, the amount of GFP-Wnt3a was increased in cell lysates of L and HEK293 cells treated with the CS of co-culture with sFRP2, but neither exFzd8 (**b**) nor FRP3 (**c**), compared to control. In each experiment, equal amounts of cell lysate were analyzed by Western blot.

Each membrane was blotted separately in **a, c**.

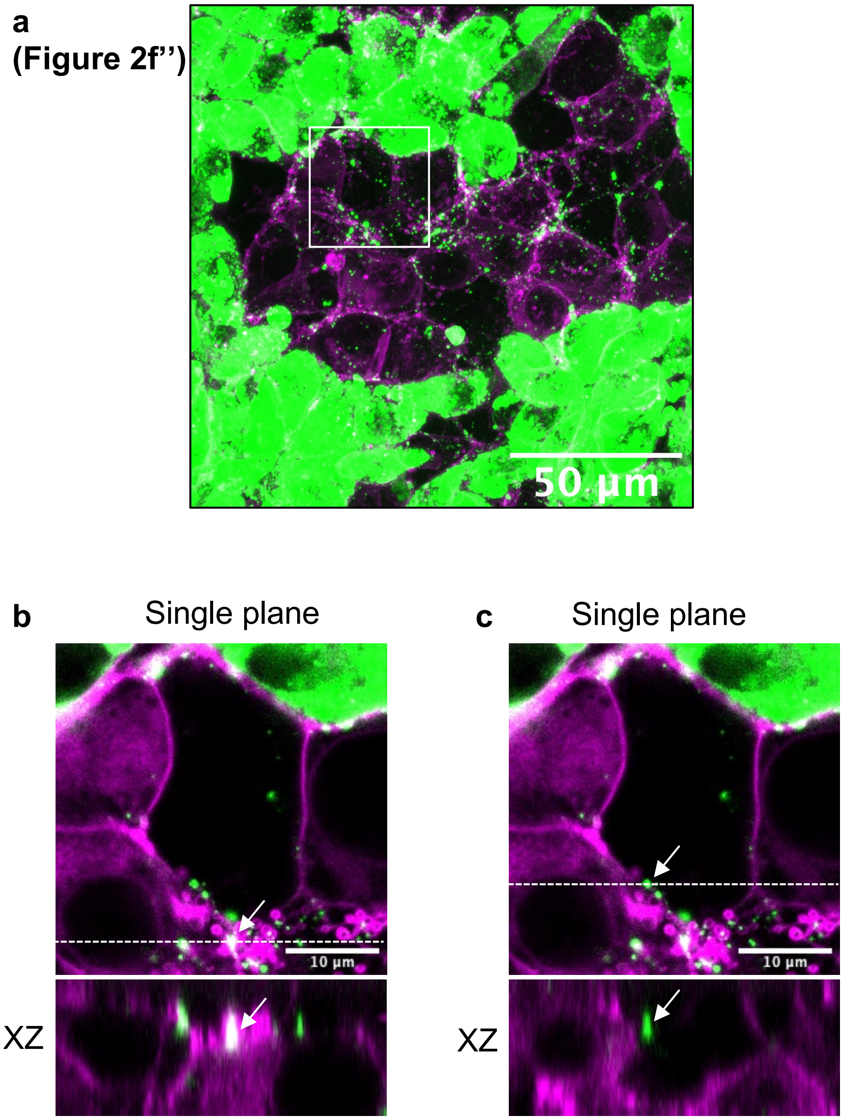

Supplementary Figure 11: Localization of GFP-Wnt3a puncta in neighboring sFRP2-expressing HEK293 cells

This figure is supplementary to Figure 2f''. Analysis of XZ planes of the area surrounded by white box (**a**) revealed that many GFP-Wnt3a puncta were localized along the cell surface (**b**) and some green puncta in (**a**) are incorporated into cells (**c**) . The white dashed lines indicate position of optical sections shown below. White arrows indicate an example of GFP-Wnt3a puncta on the cell surface (**b**) or inside the cell (**c**). Sale bar; 10 μm in (**b**) and (**c**).

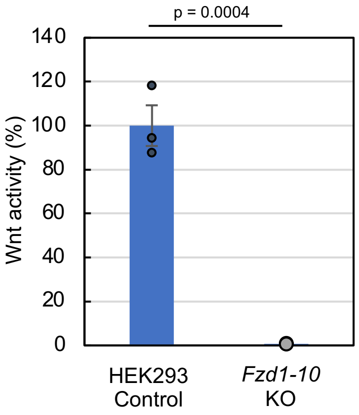

Supplementary Figure 12: Wnt/β-catenin signaling activity in *Fzd* KO cells

Wnt3a/β-catenin activity of *Fzd1-10* KO cells was monitored by transfection of plasmids expressing SuperTopFlash reporter and *Renilla* luciferase. Reporter activity was normalized to *Renilla* luciferase activity and standardized to the activity in parental HEK293 cells (n = 3). Data are expressed as means ± s.e. $p < 0.05$ was considered statistically significant, using two-sided Student's t test.

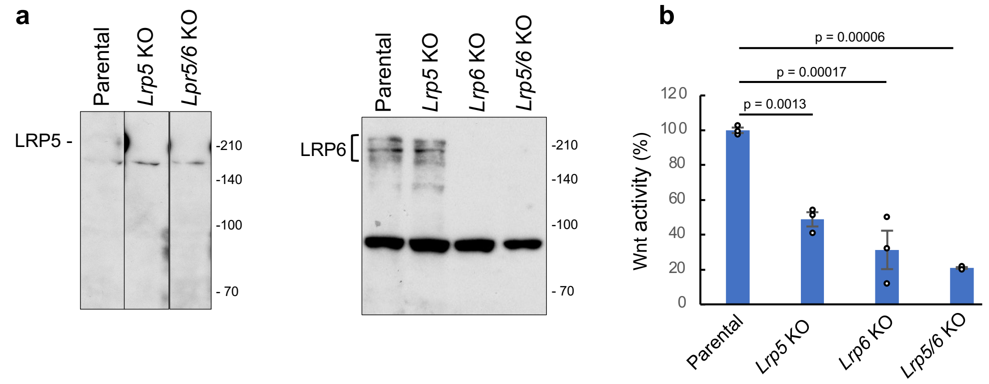

Supplementary Figure 13: Generation of *Lrp5* and *Lrp6* double KO MDCK cells

a. *Lrp5* and *Lrp6* double knock-out MDCK cells were generated by genome editing with CRISPR/Cas9 and the lack of these proteins was confirmed by Western blot analysis. LPR5 and LRP6 proteins were undetectable in double KO cells.

b. Wnt3a/β-catenin signaling activity in *Lrp5*-KO, *Lrp6*-KO, or *Lrp5/6* double KO was monitored by transfection of plasmids expressing SuperTopFlash reporter and *Renilla* luciferase. Reporter activity was normalized to *Renilla* luciferase activity and standardized to the activity in parental MDCK cells. n = 3 per each cell lines. Data are expressed as means ± s.e. $p < 0.05$ was considered statistically significant by one-way ANOVA followed by Turkey HSD test. Data are expressed as means ± s.e.

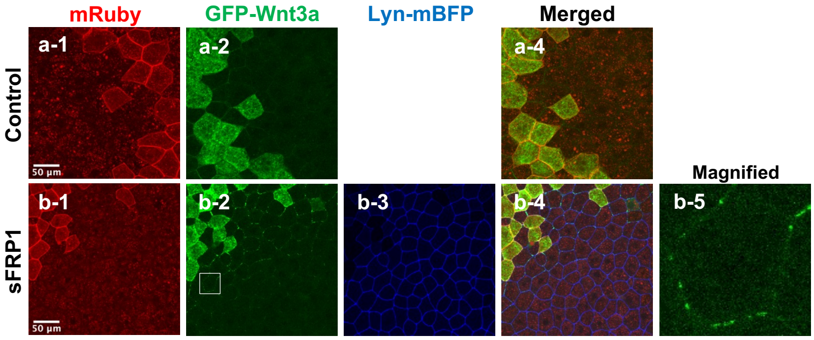

Supplementary Figure 14: Effect of sFRP proteins on the distribution of GFP-Wnt3a on surfaces of neighboring cells

This figure is supplementary to Figure 4. GFP-Wnt3a mRNA and sFRP1 mRNA were injected into different blastomeres at the four-cell stage of *Xenopus* embryos. Injected embryos were fixed with MEMFA at stage 11.5. GFP-Wnt3a-expressing cells are marked by membrane-bound Ruby (mRuby) expression (red), while neighboring cells expressing sFRP1 are identified by Lyn-mBFP expression (blue).

Injection with sFRP1 (**b**) increases GFP-Wnt3a accumulation on the cell surface, compared to controls (**a**). A magnified image of the area surrounded by a white line in **b-2** is also shown (**b-5**). Scale bar; 50 μm.

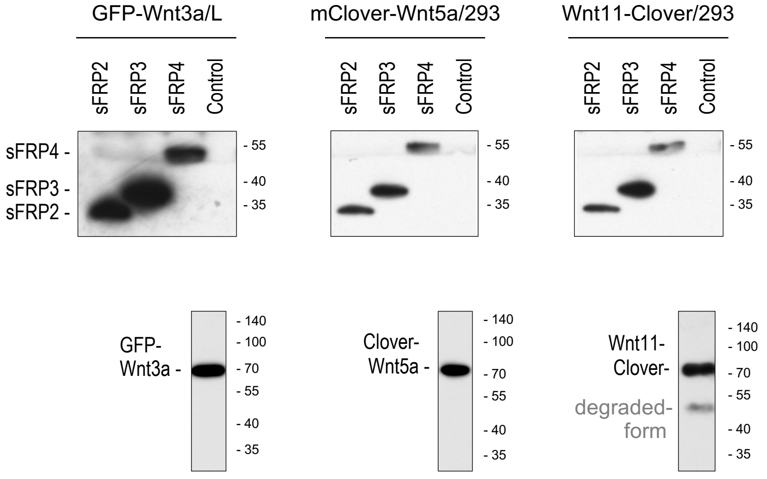

Supplementary Figure 15: Amounts of sFRP proteins in CSs subjected to AUC.

This figure is supplementary to Figure 5. Results of Western blotting analyses detecting FLAG tagged sFRPs for CSs of GFP-Wnt3a-expressing L cells, mClover-Wnt5a-expressing HEK293 cells, or Wnt11-mClover-expressing HEK293 cells co-cultured with sFRP2-, sFRP3-, sFRP4-expressing, as well as control, HEK293 cells are indicated. Wnt proteins in CM were also analyzed by Western blotting after concentration and purification by Blue-Sepharose. Note that Wnt11-Clover is partially degraded in CM. Each membrane was blotted separately.

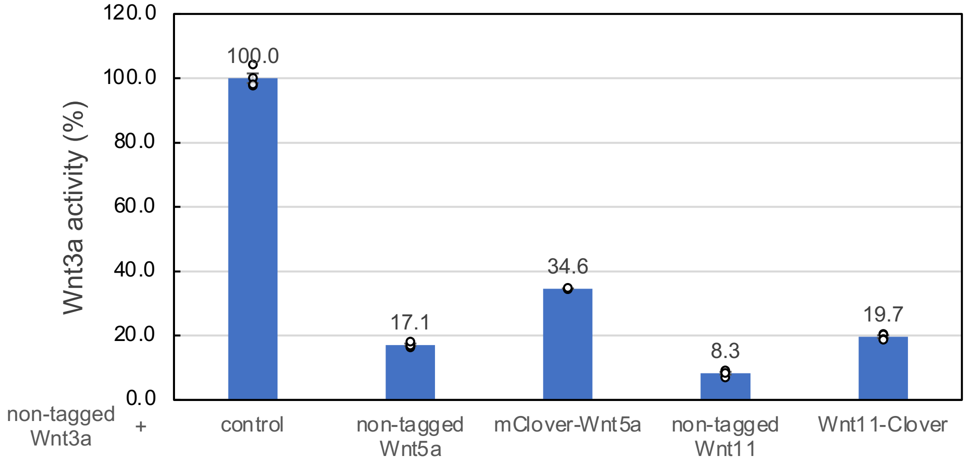

Supplementary Figure 16: Activities of tagged Wnt5a and Wnt11 proteins

This figure is supplementary to Figure 5. Activity of mClover-Wnt5a, and Wnt11-mClover are monitored by their abilities to inhibit Wnt3a/β-catenin activity. CSs of non-tagged Wnt5a, mClover-Wnt5a, non-tagged Wnt11 and Wnt11-mClover were mixed with the CS of non-tagged Wnt3a, and applied to STF/HEK293 cells. Then, luciferase activity in the lysate was measured, indicating that signaling activity remains in these constructs, although their activities per protein were reduced compared to non-tagged proteins. Activity of GFP-Wnt3a has already been reported [33, 75]. Data are expressed as means ± s.e, n = 4.

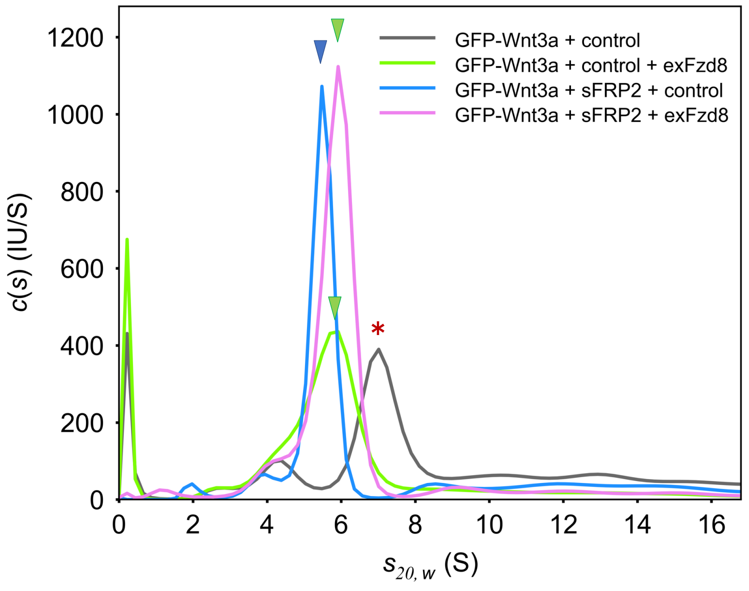

Supplementary Figure 17: AUC analysis of GFP-Wnt3a and sFRP2 with the extracellular domain of Frizzled8

By AUC-FDS analysis, GFP-Wnt3a/sFRP2 interaction was compared with or without cells expressing the extracellular domain of Frizzled8 (exFzd8). AUC-FDS analyses were conducted using the CSs of GFP-Wnt3a-expressing L cells cocultured with sFRP2-expressing or parental HEK293 cells, and also with exFzd8-expressing or parental HEK293 cells, as shown in the footnote of the figure. Ratios of inoculated cells are 8:1:1, respectively. In each case, CS was prepared 2 days after inoculation. A peak indicated by red asterisk corresponds that of the GFP-Wnt3a/afamin complex. Note that the peak corresponding to GFP-Wnt3a/sFRP2 heterodimerization (blue arrowhead) was shifted to corresponding to GFP-Wnt3a/exFzd8 (green arrowheads) in the presence of exFzd8-expressing HEK293 cells.

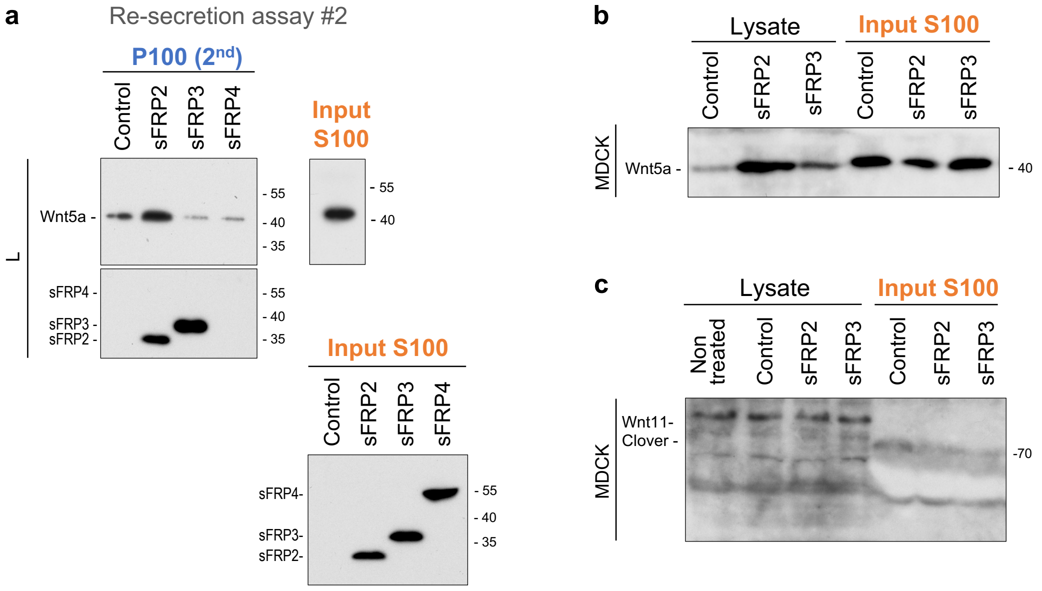

Supplementary Figure 18: Exosome-mediated Wnt5a re-secretion from L cells is increased specifically by sFRP2.

This figure is supplementary to Figure 6.

a. Exosome-mediated re-secretion of Wnt5a from L cells. The experiment shown in Figure 4a (Re-secretion assay #2) was carried out. Western blotting shows Wnt5a recovered in the P100 (2nd) fraction prepared from L cells treated with a mixture of exosome-depleted CS (S100) of non-tagged Wnt5a/L and those of sFRP2-, sFRP3-, or sFRP4-expressing, as well as control, HEK293 cells in a 1:3 ratio. Each membrane was blotted separately.

b, c. Amounts of Wnt5a (**b**) and Wnt11 (**c**) in the lysate of MDCK cells treated with mixture of exosome-depleted CS (S100) of non-tagged Wnt5a/L or Wnt11-mClover/HEK293 cells with those of sFRP2- or sFRP3-expressing, as well as control, HEK293 cells. Cell lysates were prepared 2 h after treatment of CSs.

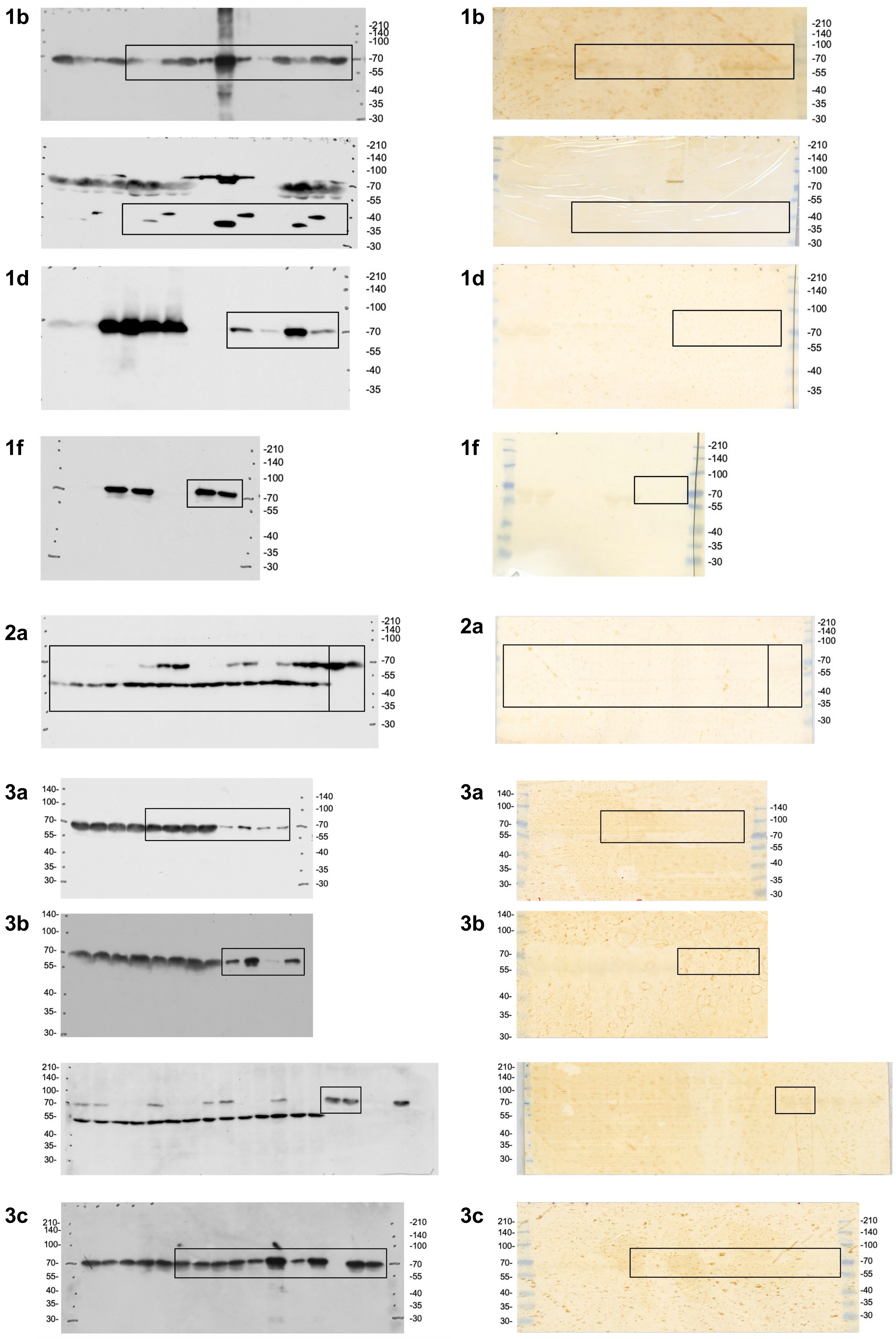

Supplementary Figure 19-1: Full blot images and corresponding blotted filters for Figure 1, 2, and 3

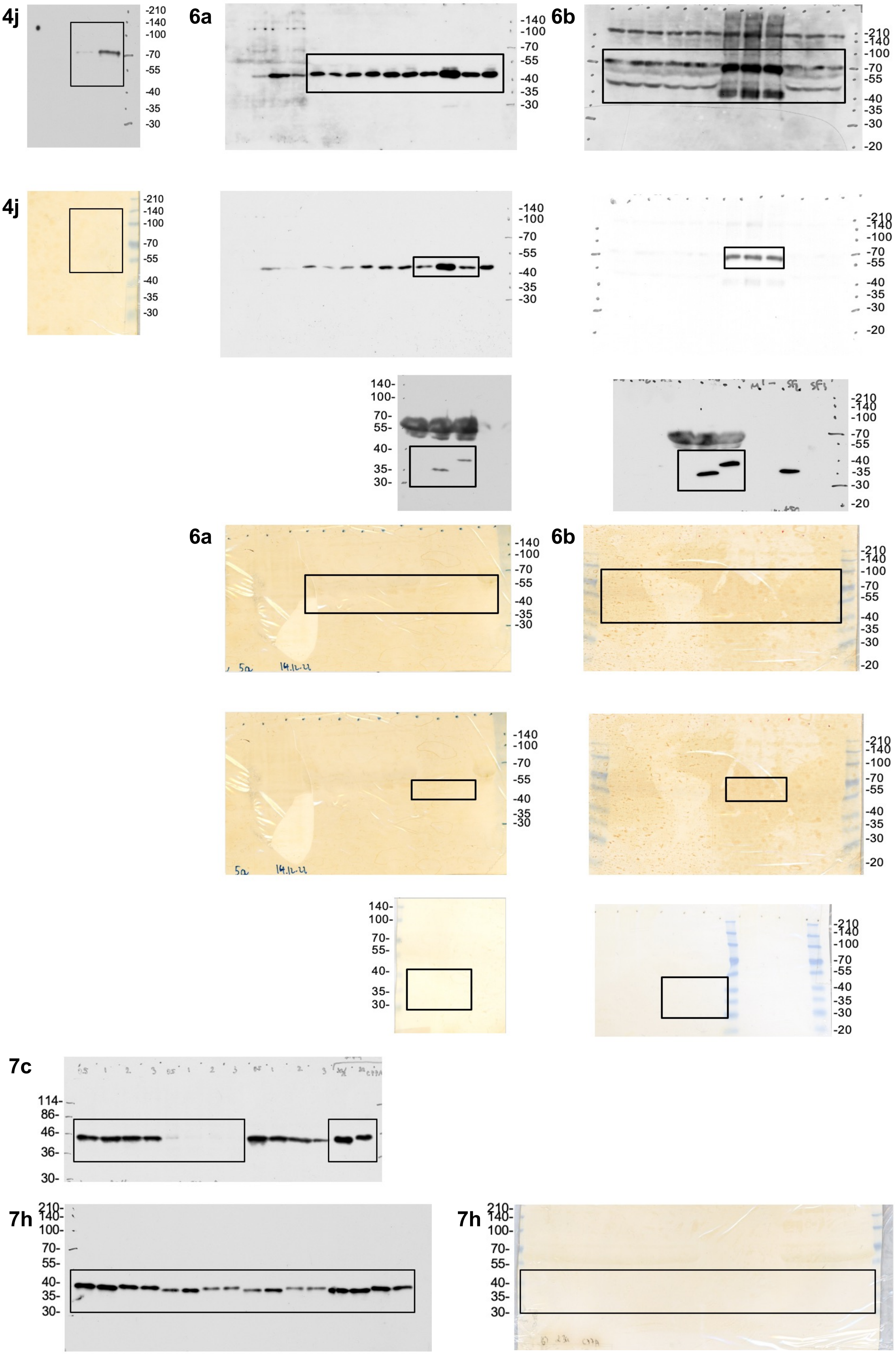

Supplementary Figure 19-2: Full blot images and corresponding blotted filters for Figure 4, 6, and 7

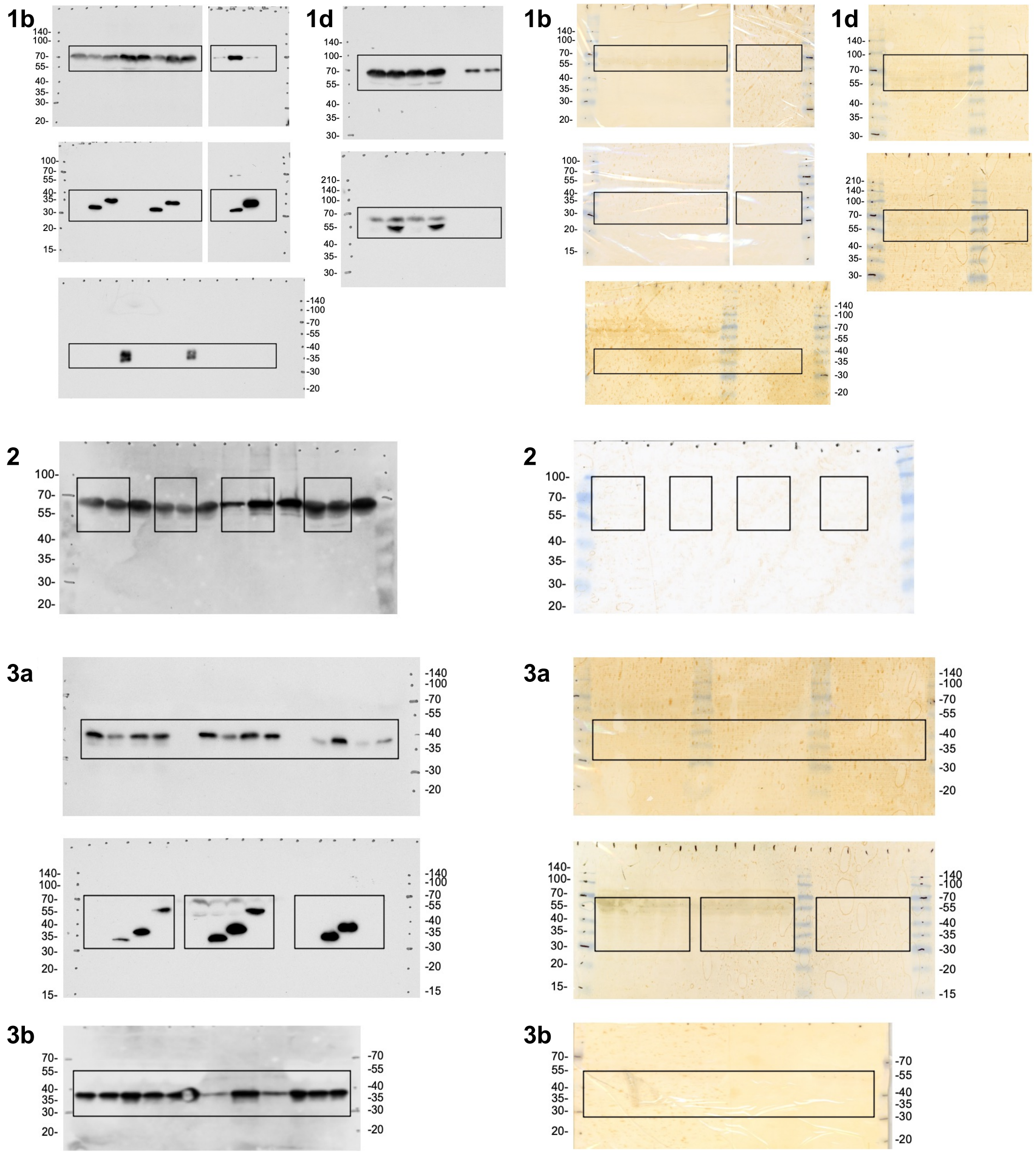

Supplementary Figure 19-3: Full blot images and corresponding blotted filters for Supplementary Figure 1, 2, and 3

4b

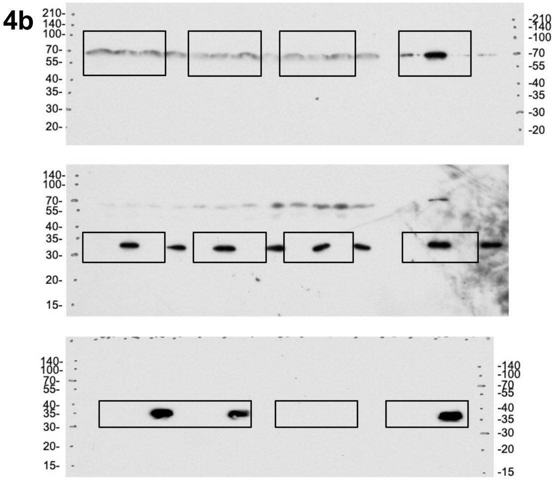

4b

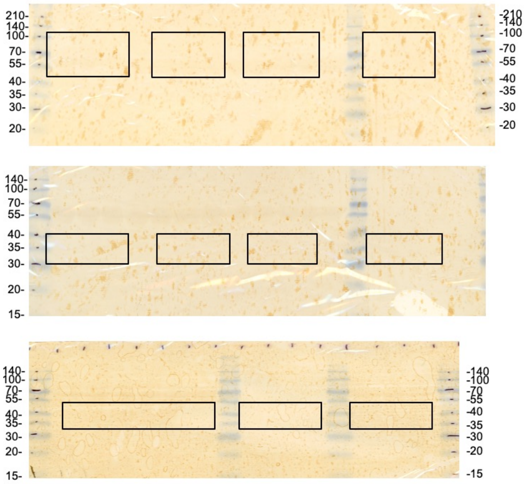

4c

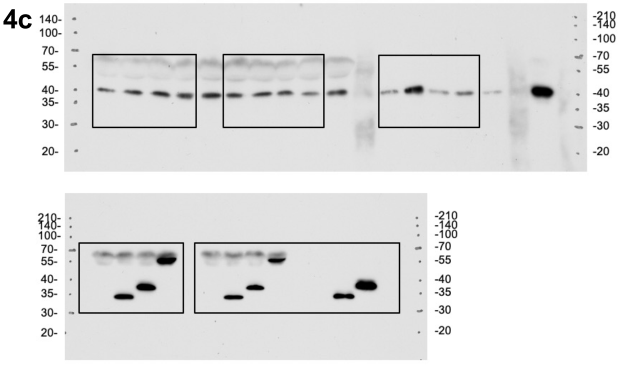

4c

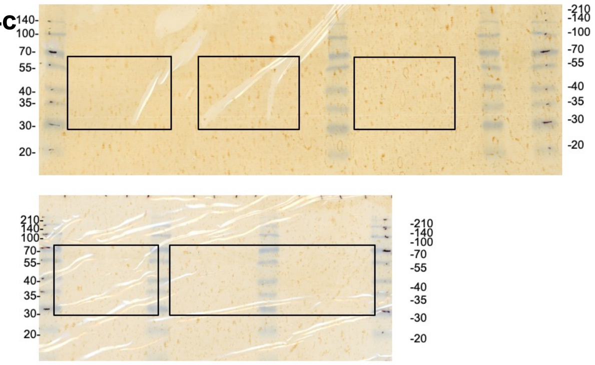

5

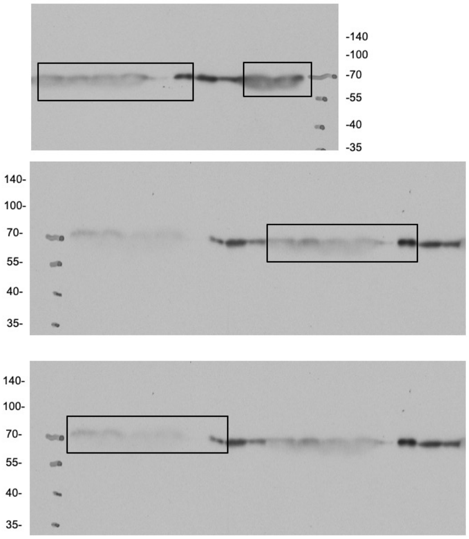

5

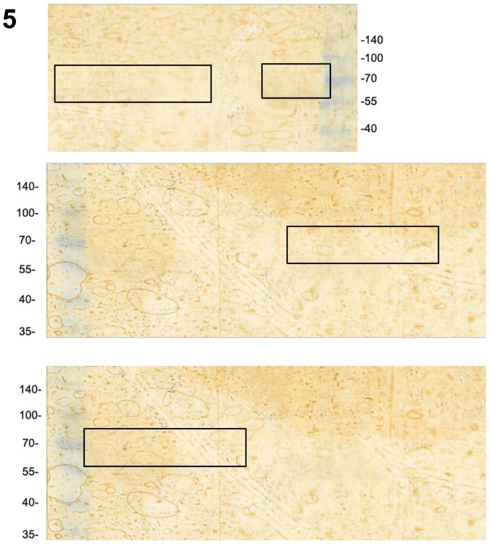

6a

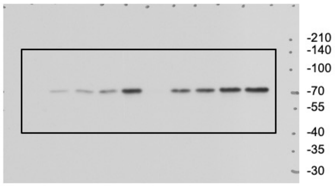

6a

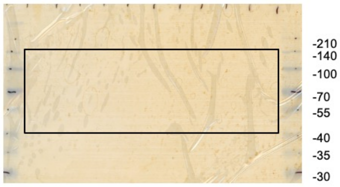

Supplementary Figure 19-4: Full blot images and corresponding blotted filters for Supplementary Figure 4, 5, and 6

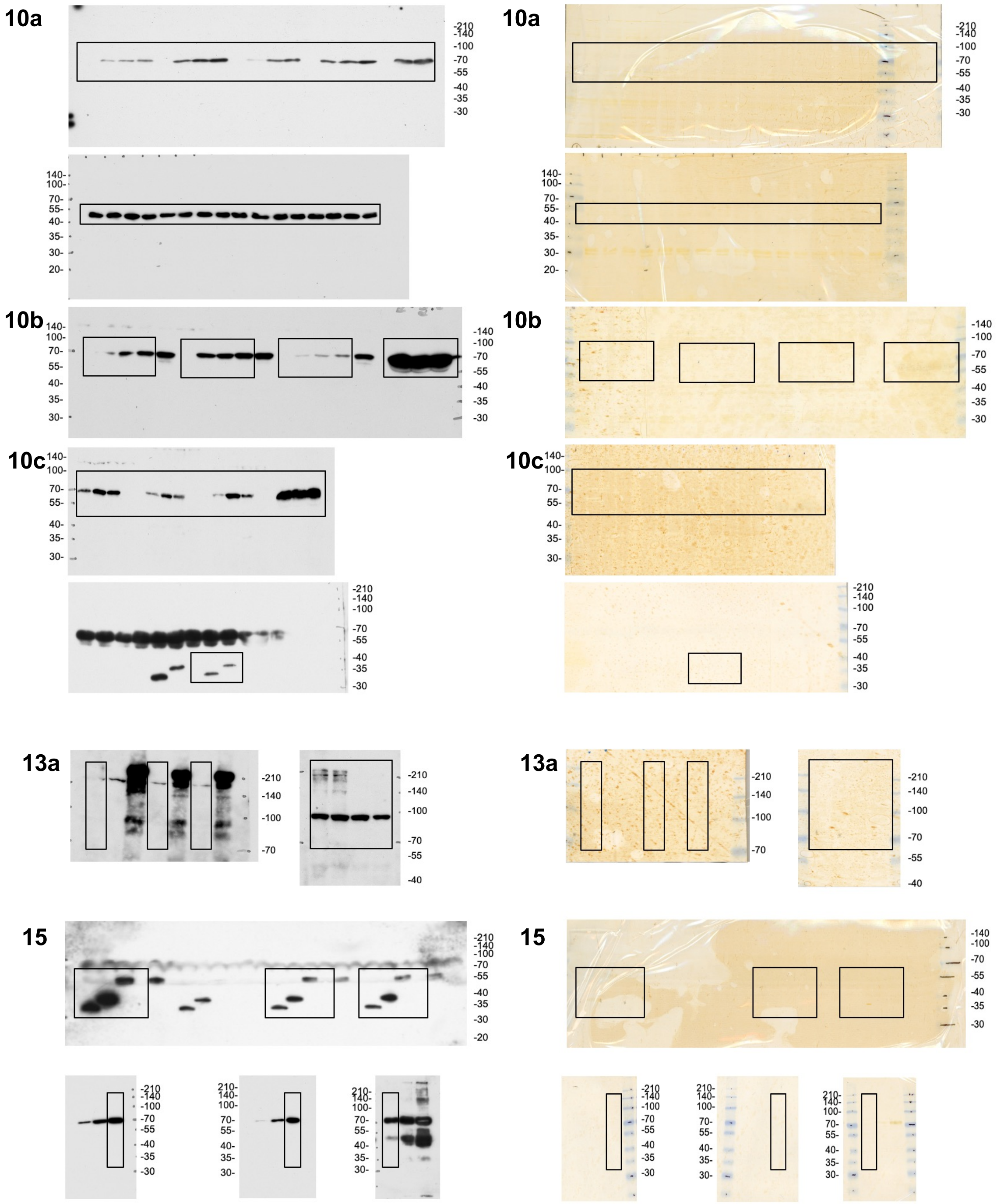

Supplementary Figure 19-5: Full blot images and corresponding blotted filters for Supplementary Figure 10, 13, and 15

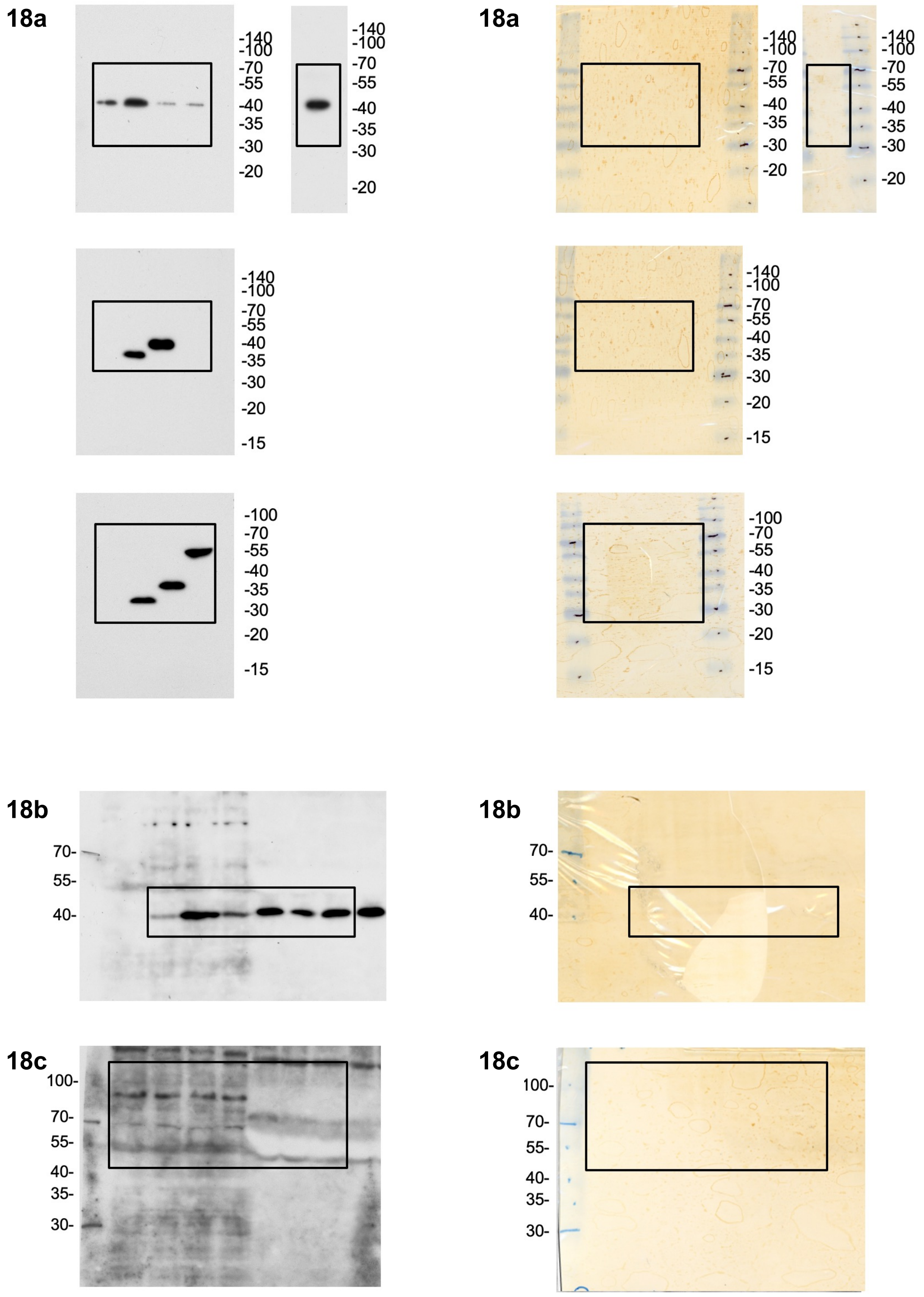

Supplementary Figure 19-6: Full blot images and corresponding blotted filters for Supplementary Figure 18

Supplementary Movie 1: Wnt3a visualization in sFRP2 cells co-expressing pHluorin-M153R-CD63-mTagBFP. mCherry-Wnt3a was co-cultured with HEK293 cells double-expressing sFRP2 and pHluorin-M153R-CD63-mTagBFP at a ratio 3:2 for 1 day before observation. Time-course images were taken at 3-min intervals.

Supplementary Movie 2: Effect of sFRP2 on dynamics of GFP-Wnt3a in co-culture of GFP-Wnt3a/L and sFRP2/HEK293. GFP-Wnt3a-producing L cells and sFRP2-producing HEK293 cells were co-cultured at a ratio of 6:4 for 2 days before observation. Cells were treated with FM 4-64 FX in final concentration of 5 µL/mL. Time-lapse images were taken every minute from treatment time. An overall image of a colony of sFRP2-expressing HEK293 cells surrounded by GFP-Wnt3a-expressing cells is shown.

Supplementary Movie 3: Movement of GFP-Wnt3a puncta incorporated into the sFRP2-expressing HEK293 cell. Magnification of Supplementary Movie 2 reveals the incorporation of GFP-Wnt3a puncta into one sFRP2-expressing HEK293.

Supplementary Table 1

Antibody (Ab) used for Western blotting	Origin
**anti-mouse Wnt3a, mouse monoclonal Ab**	Generated by Takada et al.<br>1:10 dilution
**anti-mouse Wnt5a, mouse monoclonal Ab**	Generated by Takada et al.<br>1:2 dilution
**anti-human Wnt11, rabbit polyclonal Ab**	Abcam: ab31962<br>1:1000 dilution
**anti-FLAG® M2-Peroxidase (HRP), mouse monoclonal Ab**	Sigma-Aldrich:A-8592<br>1:500 dilution
**anti-LRP5, rabbit monoclonal Ab**	Cell signaling: #2560, clone C5C7<br>1:500 dilution
**anti-LRP6, rabbit monoclonal Ab**	Cell signaling: #3395, clone C47E12<br>1:500 dilution
**anti-His-probe, rabbit polyclonal Ab**	Santacruz:sc804, #G-18<br>1:4000 dilution
**anti-HA, rat monoclonal Ab**	ROCHE: 11867423001, clone 3F10<br>1:1000 dilution
