# Supplementary material for: Soluble Frizzled-related proteins promote exosome-mediated Wnt re-secretion
Source: Commun Biol. 2024 Mar 1;7:254. doi: 10.1038/s42003-024-05881-8 (PMC10907715; doi:10.1038/s42003-024-05881-8)
Supplement: Supplementary file 2 — Description of Additional Supplementary Files [file 42003_2024_5881_MOESM2_ESM.pdf]

Description of Additional Supplementary Files

File name: Supplementary Data 1
Description: Numerical source data in the paper

File name: Supplementary Data 2
Description: Full blot images of Main Figures

File name: Supplementary Data 3
Description: Full blot images of Supplementary Figures

File name: Supplementary Movie 1
Description: Wnt3a visualization in sFRP2 cells co-expressing pHluorin-M153R-CD63-mTagBFP

File name: Supplementary Movie 2
Description: Effect of sFRP2 on dynamics of GFP-Wnt3a in co-culture of GFP-Wnt3a/L and sFRP2/HEK293
